# A Novel Parameter Estimation Method Based on a Tuneable Sigmoid in Alpha-Stable Distribution Noise Environments

**DOI:** 10.3390/s18093012

**Published:** 2018-09-08

**Authors:** Li Li, Nicolas H. Younan, Xiaofei Shi

**Affiliations:** 1 College of Information Engineering, Dalian University, Dalian 116622, China; 2 Department of Electrical and Computer Engineering, Mississippi State University, Starkville, MS 39762, USA; shixiaofei@dlmu.edu.cn; 3 Information Science and Technology College, Dalian Maritime University, Dalian 116026, China

**Keywords:** alpha stable distribution noise, tuneable Sigmoid transform, fractional power spectrum density, LFM pulse radar, parameter estimation

## Abstract

In this paper, a novel method, that employs a fractional Fourier transform and a tuneable Sigmoid transform, is proposed, in order to estimate the Doppler stretch and time delay of wideband echoes for a linear frequency modulation (LFM) pulse radar in an alpha-stable distribution noise environment. Two novel functions, a tuneable Sigmoid fractional correlation function (TS-FC) and a tuneable Sigmoid fractional power spectrum density (TS-FPSD), are presented in this paper. The novel algorithm based on the TS-FPSD is then proposed to estimate the Doppler stretch and the time delay. Then, the derivation of unbiasedness and consistency is presented. Furthermore, the boundness of the TS-FPSD to the symmetric alpha stable (SαS) noise, the parameter selection of the TS-FPSD, and the feasibility analysis of the TS-FPSD, are presented to evaluate the performance of the proposed method. In addition, the Cramér–Rao bound for parameter estimation is derived and computed in closed form, which shows that better performance has been achieved. Simulation results and theoretical analysis are presented, to demonstrate the applicability of the forgoing method. It is shown that the proposed method can not only effectively suppress impulsive noise interference, but it also does not need a priori knowledge of the noise with higher estimation accuracy in alpha-stable distribution noise environments.

## 1. Introduction

The joint estimation of the Doppler and time delay of a noise-contaminated signal is a fundamental problem in radar and sonar systems and this has been extensively addressed for the case involving narrowband signals [1,2,3,4,5,6]. Weiss [7], Remley [8], and Qu [9] indicated that the narrowband model and the corresponding narrowband signal processing techniques are applicable when BT≪c/2v, where B is the bandwidth of the transmitted signal, T is the duration of the transmitted signal, v is the relative velocity between the target and the sensor, and c is the propagation speed of the signal. The wideband signals, such as linear frequency modulation (LFM) signals with a large time frequency–bandwidth product, are frequently used in sonar and radar systems because of their lower probability of interception. In many modern radar systems, however, a wideband signal is utilized, and the narrowband model is not appropriate. In applications where BT≪c/2v is invalid, the wideband model has to be employed. In wideband radar systems, the echo often contains a Doppler stretch (DS), and not a Doppler shift only, which results in difficulty in the parameters’ estimation. For the determination of the range and the relative velocity of the target, the accurate estimation of these parameters is crucial. 

Since the fractional Fourier transform (FRFT) spectrum of a LFM signal has a greater energy concentration characteristic, the FRFT, as a new time–frequency tool, has attracted more attention, and has been widely applied to the parameter estimation of LFM signal [10,11,12,13,14,15,16]. Zhao et al. propose a method for estimating the LFM signal by utilizing the pulse compression in both the time domain and the FRFT domain [10]. The parameters of the LFM signal can be estimated by a fractional Fourier transform [11,12], a fractional correlation [13], and a fractional power spectrum [14,15,16].

Up to now, in most parameter estimation methods for array signal processing, the additive noise is assumed to be Gaussian. Studies and experimental measurements have shown that a broad class of noise such as underwater acoustic noise, atmospheric noise, multiuser interference, and radar clutters in real world applications are non-Gaussian, primarily owing to impulsive phenomena [17,18]. Taking these scenarios into account, it is inappropriate to model the noise as Gaussian noise. Researchers have studied this impulsive nature, and shown that the symmetric alpha stable (SαS) processes are better models for impulsive noise than the Gaussian processes. The conventional algorithms based on second-order statistics degenerate severely in the SαS noise environment. 

To reduce the SαS noise interference, many parameter estimation algorithms based on the fractional lower-order statistics (FLOS) have been proposed [16,19,20,21]. However, these algorithms do need a priori knowledge of the SαS noise and have other limitations, where the characteristic exponent α and the fractional lower order of moments p must meet the need of 1≤p<α or 0<p<α/2, otherwise those algorithms performance can degrade seriously, or even become invalid, while the fractional lower-order moment value is not appropriate.

The Sigmoid function is widely used as a common nonlinear transform. The Sigmoid function can suppress impulsive noise interference, and this does not depend on a priori knowledge of the noise. Yu et al., propose a method based on generalized Sigmoid cyclic cross-ambiguity function to estimate the time delay and Doppler frequency shift in the impulsive noise and co-channel interference [22]. In [22], the signal is assumed to be the real signal. However, when the signal is a complex signal, the results do not always hold.

To handle this problem, a novel concept termed the tuneable Sigmoid transform fractional correlation (TS-FC) is proposed in this paper, and a relative method named the tuneable Sigmoid fractional power spectrum density (TS-FPSD) is presented, to fulfill the needs mentioned above. The novel algorithm based on the TS-FPSD is then proposed to estimate the Doppler stretch and time delay. In addition, we address unbiasedness and consistency by adding their corresponding derivation. Furthermore, the boundness of the TS-FPSD to the SαS noise, the parameter selection of the TS-FPSD, the feasibility analysis of the TS-FPSD, and the Cramér–Rao bound for parameter estimation are presented, to evaluate the performance of the proposed method. The proposed method does not need a priori knowledge of the alpha stable distribution noise.

This paper is organized as follows. Section 2 presents a signal model of wideband echoes in an alpha-stable distribution noise environment. In Section 3, the analysis of the fractional power spectrum density is presented. In Section 4, a novel tuneable Sigmoid fractional correlation function (TS-FC) and a novel tuneable Sigmoid fractional power spectrum density function (TS-FPSD) are defined. In addition, unbiasedness and consistency are derived. In Section 5, a novel Doppler stretch and time delay estimation method based on TS-FPSD for the SαS noise is proposed. In addition, the boundness of the TS-FPSD to the SαS noise, parameter selection of the TS-FPSD, and a feasibility analysis of the TS-FPSD are analyzed, and the Cramér–Rao bound for parameter estimation is derived. In Section 6, the performance of the parameter estimation algorithm is studied through extensive numerical simulations. Finally, conclusions are drawn in Section 7.

## 2. The Signal Model and the Noise Model

### 2.1. The Signal Model

We consider x(t) as a LFM signal, defined as:

(1) $$x\left(t\right)=\left\{ \begin{array}{l} A\exp\left(j2\pi \left(f_{0}t+\frac{1}{2}\mu_{0}t^{2}\right)\right),\;0<t<T\\ 0,\;T<t<T_{0} \end{array}\right.\;$$

where $f_{0}$ and $\mu_{0}$ are the initial frequency and the frequency modulation rate, respectively. A denotes the signal amplitude, $T_{0}$ denotes the modulation period, and T denotes the LFM pulse duration. In a wideband radar system, the echo from a wideband signal often contains a Doppler stretch (DS), and not a Doppler shift only. The echo y(t) can be expressed as [9]:

(2) $$\begin{array}{cc} y\left(t\right) & =\sum_{l=1}^{L}\beta_{l}x\left(\sigma_{l}\left(t-\tau_{l}\right)\right)+n\left(t\right)\\ & =\left\{ \begin{array}{l} \sum_{l=1}^{L}\beta_{l}A\exp\left(j2\pi \left(\sigma_{l}f_{0}\left(t-\tau_{l}\right)+\frac{1}{2}\mu_{0}\sigma_{l}^{2}{\left(t-\tau_{l}\right)}^{2}\right)\right)+n\left(t\right),\;0\leq t\leq T\\ 0\;T\leq t\leq T_{0} \end{array}\right. \end{array}$$

where $\beta_{l}$ denotes the attenuation factor of the *l*th multipath,$\sigma_{l}$ is the Doppler stretch, $\tau_{l}$ is the time delay (TD), and L denotes the number of the echo. The noise n(t) is a sequence of the independent and identically distributed (i.i.d) isotropic complex SαS random variable.

### 2.2. The SαS Distribution Noise Model

In order to evaluate the robustness of the proposed method, the α-stable distribution noise is modeled by a complex isotropic symmetric α-stable (SαS) noise distribution [16,17]. The characteristic function of the SαS distribution is defined as follows:

(3) $$\rho \left(\omega \right)=\exp\left(-\gamma {\left|\omega \right|}^{\alpha }\right)\;$$

where α(0<α≤2) is usually called the characteristic exponent. When α<2, the distribution is algebraic-tailed with a tail constant α, implying infinite variance. The smaller it is, the heavier the tails of the density. When *α* = 2, the *SαS* distribution reduces to the Gaussian distribution. The parameter γ, usually called the dispersion, is a positive constant that is related to the scale of the distribution. The parameter γ plays a role that is analogous to that of the variance for a second-order process. The following proposition gives us a closed-form expression for the geometric power of the symmetric α-stable random variables [18]:

(4) $$S_{0}={\left(C\gamma \right)}^{1/\alpha }/C\;$$

where C≈1.78.

Since the α-stable distribution with α<2 determines an infinite variance, we describe the signal-to-noise condition of SαS using the generalized signal-noise-ratio (GSNR) [17], which is defined as:

(5) $$\mathrm{GSNR}=10\mathrm{lg}\left(\sigma_{x}^{2}/\gamma \right)\;$$

where $\sigma_{x}^{2}$ and γ are the variance of the underlying signal and dispersion of the SαS noise, respectively.

## 3. Analysis of the Fractional Power Spectrum Density

### 3.1. The Fractional Correlation Function and the Fractional Power Spectrum Density

The fractional Fourier transform (FRFT) is a generalization of the FT, and it can be interpreted as a rotation of the signal to any angles in the time–frequency plane [15,16].

The fractional correlation function (FC) of the signal x(t) is defined as [15]:

(6) $${\hat{R}}_{xx}^{\rho }\left(\xi \right)=\underset{T\to \infty }{\lim}\frac{1}{2T}\int_{-T}^{+T}R_{xx}\left(t+\xi ,t\right)\exp\left(jt\xi \cot\rho \right)dt\;$$

where $R_{xx}\left(t+\xi ,t\right)$ is the correlation function of the signal x(t), ξ denotes the delay, and ρ denotes the rotation angle in the FRFT domain.

The fractional power spectrum density function (FPSD) of the signal x(t) is expressed as [15]:

(7) $$P_{xx}^{\rho }\left(m\right)=A_{-\rho }F^{\rho }\left[{\hat{R}}_{xx}^{\rho }\left(\xi \right)\right]\left(m\right)\exp\left(-jm^{2}\cot\rho /2\right)\;$$

where $A_{\rho }=\sqrt{\frac{1-j\cot\rho }{2\pi }}$, m denotes the frequency in FRFT domain, and $F^{\rho }\left[\cdot \right]\left(m\right)$ denotes the fractional Fourier transform.

The continuous FRFT [11] of a signal x(t) with a rotation angle ρ, is defined as:

(8) $$X\left(\rho ,m\right)=F^{\rho }\left[x\left(t\right)\right]\left(m\right)=\int_{-\infty }^{+\infty }x\left(t\right)K_{b}\left(t,m\right)dt\;$$

where $F^{b}$ denotes the FRFT operator, b(0<b≤2) denotes the fractional order, ρ≡bπ/2, and $K_{b}\left(t,m\right)$ is the kernel function of the fractional Fourier transform. $K_{b}\left(t,m\right)$ can be expressed as:

(9) $$K_{b}\left(t,m\right)=\left\{ \begin{array}{l} A_{\rho }\exp\left(\frac{j}{2}\left(t^{2}\cot\rho -2mt\csc\rho +m^{2}\cot\rho \right)\right),\;\rho \neq n\pi \\ \delta \left(t-m\right),\;\rho =2n\pi \\ \delta \left(t+m\right),\;\rho =\left(2n+1\right)\pi \end{array}\right.\;$$

### 3.2. The Parameter Estimation Based on the Fractional Power Spectrum Density Function

From Equations (2) and (6), the fractional correlation ${\hat{R}}_{yy}^{\rho }\left(\xi \right)$ of the echo signal y(t) can be expressed as (see Appendix A for details):

(10) $$\begin{array}{cc} {\hat{R}}_{yy}^{\rho }\left(\xi \right) & =\sum_{l=1}^{L}\underset{T\to \infty }{\lim}\frac{1}{2T}\int_{-T}^{+T}E\left[y\left(t+\xi \right)y_{}^{*}\left(t\right)\right]exp\left(jt\xi \cot\rho \right)dt\\ & =\sum_{l=1}^{L}\beta_{l}^{2}A^{2}\exp\left(j2\pi \left(f_{0}\sigma_{l}-\mu_{0}\tau_{l}\sigma_{l}^{2}\right)\xi +\frac{1}{2}\mu_{0}\sigma_{l}^{2}\xi^{2}\right)\underset{T\to \infty }{\lim}\frac{1}{2T}\int_{-T}^{+T}dt\int_{-T}^{+T}\exp\left(j\left(2\pi \mu_{0}\sigma_{l}^{2}+\cot\rho \right)t\xi \right)dt\\ & +{\hat{R}}_{yy,lq}^{\rho }\left(\xi \right)+{\hat{R}}_{yn}^{\rho }\left(\xi \right)+{\hat{R}}_{nn}^{\rho }\left(\xi \right) \end{array}$$

where ${\hat{R}}_{yy,lq}^{\rho }\left(\xi \right)$ denotes the fractional correlation function between $y_{l}\left(t\right)$ and $y_{q}\left(t\right)$, ${\hat{R}}_{yn}^{\rho }\left(\xi \right)$ denotes the fractional correlation functions between the echo signal y(t) and the noise n(t), and ${\hat{R}}_{yn}^{\rho }\left(\xi \right)$ is treated as a random interference. ${\hat{R}}_{nn}^{\rho }\left(\xi \right)$ denotes the fractional correlation function of the noise n(t).

When $2\pi \mu_{0}\sigma_{l}^{2}=-\cot\rho_{l}$, Equation (10) can be rewritten as:

(11) $${\hat{R}}_{yy}^{\rho_{l}}\left(\xi \right)=\sum_{l=1}^{L}\beta_{l}^{2}A^{2}\exp\left(j2\pi \left(f_{0}\sigma_{l}-\mu_{0}\tau_{l}\sigma_{l}^{2}\right)\xi +\frac{1}{2}\mu_{0}\sigma_{l}^{2}\xi^{2}\right)+{\hat{R}}_{yy,lq}^{\rho_{l}}\left(\xi \right)+{\hat{R}}_{yn}^{\rho_{l}}\left(\xi \right)+{\hat{R}}_{nn}^{\rho_{l}}\left(\xi \right)\;$$

From Equation (11), we can find that ${\hat{R}}_{yy}^{\rho_{l}}\left(\xi \right)$ has the expression of a LFM signal, which has the characteristics of energy that is concentrated in the FRFT domain. However, ${\hat{R}}_{yy,lq}^{\rho_{l}}\left(\xi \right)$,${\hat{R}}_{yn}^{\rho_{l}}\left(\xi \right)$, and ${\hat{R}}_{nn}^{\rho_{l}}\left(\xi \right)$ do not have such characteristics in the FRFT domain; the amplitudes of TS-FPSD of different targets are very low at $\left(\rho_{l},m_{l}\right)$, and these signals are not considered as randomly interfering. Therefore, the fractional power spectrum density $P_{yy}^{\rho }\left(m\right)$ of the echo signal y(t) can be computed as:

(12) $$\begin{array}{cc} P_{yy}^{\rho }\left(m\right) & =A_{-\rho }F^{\rho }\left[{\hat{R}}_{yy}^{\rho_{l}}\left(\tau \right)\right]\left(m\right)\exp\left(-jm^{2}\cot\rho /2\right)\\ & =\sum_{l=1}^{L}A_{-\rho }A_{\rho }\int_{-\frac{T}{2}}^{+\frac{T}{2}}\exp\left(j\left(2\pi f_{0}\sigma_{l}-2\pi \mu_{0}\tau_{l}\sigma_{l}^{2}-m\csc\rho \right)\xi +\frac{j}{2}\left(2\pi \mu_{0}\sigma_{l}^{2}+\cot\rho \right)\xi^{2}\right)d\xi +P_{N}^{\rho }\left(m\right) \end{array}$$

The $P_{yy}^{\rho }\left(m\right)$ forms a pulse in the FRFT domain, and its peak value appears at $\left(\rho_{l},m_{l}\right)$ as:

(13) $$\left\{ \begin{array}{l} \cot\rho_{l}=-2\pi \mu_{0}\sigma_{l}^{2}\\ m_{0}\csc\rho_{l}=2\pi f_{0}\sigma_{l}-2\pi \mu_{0}\sigma_{l}^{2}\tau_{l}\\ \left(\rho_{l}=-\mathrm{arc}\cot2\pi \mu_{0}\sigma_{l}^{2},m_{l}=2\pi \left(f_{0}\sigma_{l}-2\pi \mu_{0}\sigma_{l}^{2}\tau_{l}\right)\sin\beta_{0}\right)=\underset{\rho ,m}{\arg}\max\left|P_{yy}^{\rho }\left(m\right)\right| \end{array}\right.\;$$

Then, it follows directly from Equation (13) that the Doppler stretch $\sigma_{l}$ and the time delay $\tau_{l}$ are estimated by [16]:

(14) $$\left\{ \begin{array}{l} {\hat{\sigma }}_{l}=\sqrt{-\frac{\cot\rho_{l}}{2\pi \mu_{0}}}\\ {\hat{\tau }}_{l}=\frac{2\pi f_{0}{\hat{\sigma }}_{l}-m_{l}\csc\rho_{l}}{2\pi \mu_{0}{\hat{\sigma }}_{l}^{2}} \end{array}\right.\;$$

When the alpha-stable distribution noise is added, this peak location algorithm may fail. The reason is that the alpha-stable distribution does not have a finite α-order moment and other higher-than-α-order moments, and the fractional correlation function and fractional power spectrum density function are based on a second-order moment. Accordingly, the fractional power spectrum density algorithm will become unbounded when the received signal contains an alpha-stable distribution noise. Therefore, we present a tuneable Sigmoid transform, to suppress the alpha-stable distribution noise interference. 

## 4. The Tuneable Sigmoid-Based Fractional Power Spectrum Density

### 4.1. The Tuneable Sigmoid Transform

The Sigmoid function is widely used as a common nonlinear transform [23,24,25]. Its definition is shown in Equation (15):

(15) $$\mathrm{Sigmoid}\left[x\left(t\right)\right]=\frac{2}{1+\exp\left[-\lambda x\left(t\right)\right]}-1\;$$

where λ is the inclined coefficient to adjust x(t) at different scales. The parameter λ is used as a scale factor to fit various signals and noises. A proper λ will retain sufficient information regarding the time delay and Doppler stretch, and attenuate most of the outliers at the same time. Thus, the proper selection of λ will ensure the accuracy of the estimation. The selection of λ is analyzed in Section 5.3. Through the analysis, the inclined coefficient λ for the TS-FPSD is set as λ=1 in all the later simulations of this paper.

For a SαS process with a=0, the Sigmoid transform has some properties as follows [22,26]:

Property 1. If x(t) is a SαS process with β=0 and a=0, then Sigmoid[x(t)] has a symmetric distribution with zero mean in its probability density function.

Property 2. *If*x(t)*is a*SαS*process with*γ>0*and*a=0*, then*${\left\|\mathrm{Sigmoid}\left[x\left(t\right)\right]\right\|}_{\alpha }>0$*, and the mean value of*Sigmoid[x(t)] is zero.

Property 3. *If*x(t)*is a*SαS*process with*a=0*, then*Sigmoid[x(t)] has a finite second-order moment with zero mean (referred to as second order moment process).

According to the first three properties, we can derive the following property as:

Property 4. *Set*X(t)=Sigmoid[x(t)]*, then*X(t)*has the same Doppler shift as*x(t).

Proof. If we set $x_{2}\left(t\right)=x\left(ct\right)$, then we know that $X_{2}\left(t\right)=\mathrm{Sigmoid}\left[x_{2}\left(t\right)\right]=\mathrm{Sigmoid}\left[x\left(ct\right)\right]=X\left(ct\right)$.

If we set $F_{x}\left(\omega \right)$ is the Fourier transform of x(t) and $F_{X}\left(\omega \right)$ is that of X(t), they satisfy the following relationship $F_{x}\left(\omega \right)=\int_{-\infty }^{\infty }x\left(t\right)e^{-j\omega t}dt$ and $F_{X}\left(\omega \right)=\int_{-\infty }^{\infty }X\left(t\right)e^{-j\omega t}dt$. Then, we know $F_{x_{2}}\left(\omega \right)=\frac{1}{c}\int_{-\infty }^{\infty }x\left(ct\right)e^{-j\omega ct/c}dct=\frac{1}{c}F_{x}\left(\omega /c\right)$ and $F_{X_{2}}\left(\omega \right)=\frac{1}{c}\int_{-\infty }^{\infty }X\left(ct\right)e^{-j\omega ct/c}dct=\frac{1}{c}F_{X}\left(\omega /c\right)$.

The frequency shift between x(t) and $x_{2}\left(t\right)$ is the same as the frequency shift between X(t) and $X_{2}\left(t\right)$. The frequency shift could be arbitrary as the parameter *c* varies.

Therefore, X(t) has the same modulation characteristic as x(t).

Property 5. *Set*X(t)=Sigmoid[x(t)]*, then*X(t)*has the same time delay as*x(t).

Proof. Set $x_{1}\left(t\right)=x\left(t-D\right)$, then:

(16) $$\begin{array}{cc} X_{1}\left(t\right)= & \frac{2}{1+\exp\left(-x_{1}\left(t\right)\right)}-1\\ & =\frac{2}{1+\exp\left(-x\left(t-D\right)\right)}-1\\ & =X\left(t-D\right) \end{array}$$

That is to say, that the Sigmoid transform does not change the time delay contained in x(t).

Property 6. *If*x(t)*is a periodic function, then*X(t)*has the same period as*x(t).

Proof. For a periodic signal x(t), satisfying $x\left(t+T^{\prime }\right)=x\left(t\right)$, where $T^{\prime }$ is its period. Then:

(17) $$\begin{array}{cc} X\left(t+T^{\prime }\right)= & \mathrm{Sigmoid}\left[x\left(t+T^{\prime }\right)\right]=\mathrm{Sigmoid}\left[x\left(t\right)\right]\\ & =X\left(t\right) \end{array}$$

Accordingly, X(t) is periodic. Since $X\left(t\right)=\mathrm{Sigmoid}\left[x\left(t\right)\right]=\frac{2}{1+\exp\left[-x\left(t\right)\right]}-1$ is a monotonic increasing function for x(t), the period of X(t) is the same as that of x(t).

### 4.2. Definition of the Tuneable Sigmoid-FC and the Tuneable Sigmoid-FPSD

Overcoming the limitations of the performance degradation of existing methods based on the fractional Fourier transform in the alpha-stable distribution noise- and the fractional lower-order statistics-based methods depends on the priori knowledge of noise. This paper presents two novel function definitions, the TS-FC and the TS-FPSD. 

A novel fractional correlation, ${\hat{R}}_{xx,\rho }^{TS}\left(\xi \right)$, referred to as the TS-FC, is defined as:

(18) $${\hat{R}}_{xx,\rho }^{TS}\left(\xi \right)=\underset{T\to \infty }{\lim}\frac{1}{2T}\int_{-T}^{+T}R_{xx}^{\mathrm{Sigmoid}}\left(t+\xi ,t\right)\exp\left(jt\xi \cot\rho \right)dt\;$$

where $R_{xx}^{\mathrm{Sigmoid}}\left(t+\xi ,t\right)=E\left\{\mathrm{Sigmoid}\left[x(t+\xi )\right]{\mathrm{Sigmoid}}^{*}[x(t)]\right\}$.

A novel power spectrum function $P_{xx,\rho }^{TS}\left(m\right)$, referred to as the TS-FPSD, is defined as:

(19) $$P_{xx,\rho }^{TS}\left(m\right)=A_{-\rho }F^{\rho }\left[{\hat{R}}_{xx,\rho }^{TS}\left(\xi \right)\right]\left(m\right)\exp\left(-jm^{2}\cot\rho /2\right)\;$$

The TS-FC in the discrete-time case can be defined as follows [15]:

(20) $${\hat{R}}_{xx,\rho }^{TS}\left(k\right)=\underset{N\to \infty }{\lim}\frac{1}{2N+1}\sum_{n=-N}^{N}R_{xx}^{\mathrm{Sigmoid}}\left(n+k,n\right)\exp\left(jnkT^{2}\cot\rho \right)\;$$

where T is the sampling period, t=nT.

Let w=mT; the discrete-time TS-FPSD can be defined as:

(21) $$P_{xx,\rho }^{TS}\left(w\right)=A_{-\rho }{\tilde{F}}^{\rho }\left[{\hat{R}}_{xx,\rho }^{TS}\left(k\right)\right]\left(w\right)\exp\left(-j\left(w^{2}/2T^{2}\right)\cot\rho \right)\;$$

**Figure 1:**
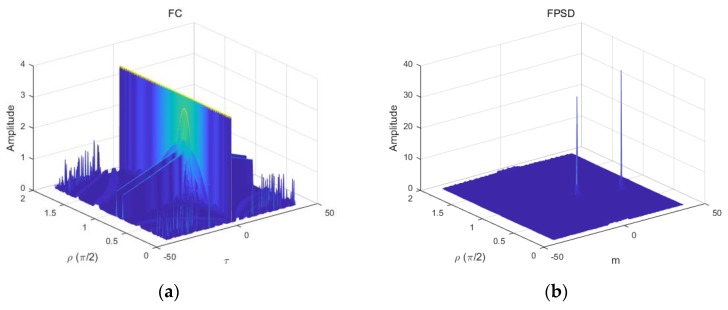

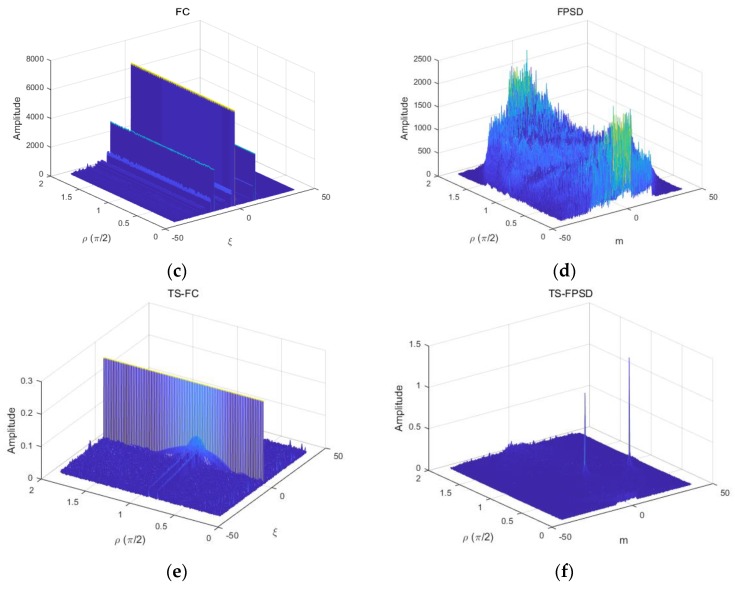
Time–frequency distribution of the fractional power spectrum density (FPSD) and tuneable Sigmoid (TS)-FPSD. (**a**) The fractional correlation (FC) of two LFM signals; (**b**) the FPSD of two LFM signals; (**c**) the FC of two LFM signals with SαS noise; (**d**) the FPSD of two LFM signals with SαS noise; (**e**) the TS-FC of two LFM signals with SαS noise; (**f**) the TS-FPSD of two LFM signals with SαS noise.

Figure 1 shows the spectrum of the FPSD and the TS-FPSD in the no-noise and impulsive noise environments, respectively. In the α-stable distribution noise environment with GSNR = 5 dB, α=1.2, and L=2, Figure 1a,b shows the time–frequency distribution of the fractional correlation (FC) and the FPSD of two LFM signals in the no-noise environment. From Figure 1a,b, we can find that the plots are smooth because there is no interference of noise. Figure 1c,d present that the time–frequency distribution of FC and FPSD of two echoes with the SαS noise. Figure 1e,f present the time–frequency distribution of the TS-FC and the TS-FPSD of two echoes with SαS noise. From Figure 1, it is clearly seen that identifying the correct peak location is not trivial, as the FPSD peak could not be distinguished from the SαS noise. Accordingly, the estimation performance of the FPSD method degraded severely in the SαS noise environment. After the application of the tuneable Sigmoid transformation, the SαS noise was suppressed effectively, and the TS-FPSD spectrum formed an obvious pulse in the FRFT domain. Thus, the method based on the TS-FPSD yielded better estimation performance. The Sigmoid transform can result in some information loss, same as other nonlinear transforms, but its capability for suppression impulsive noise is employed in this paper.

### 4.3. Unbiasedness and Consistency

In statistical theory, the bias (or bias function) of an estimator is the difference between the estimator’s expected value and the true value of the parameter being estimated. In general, the bias is related to consistency, where consistent estimators are convergent and asymptotically unbiased (hence they converge to the correct value as the number of data points grows arbitrarily large) [27,28].

In this section, the unbiasedness and consistency of the TS-FC and TS-FPSD are analyzed, to evaluate the performance of the proposed method. The derivation of the unbiasedness and consistency are presented in details in Appendix B.

#### 4.3.1. Unbiasedness and Consistency of the TS-FC 

Let the estimator of the TS-FC ${\hat{\hat{R}}}_{xx,\rho }^{TS}\left(k\right)$ is:

(22) $${\hat{\hat{R}}}_{xx,\rho }^{TS}\left(k\right)=\underset{N\to \infty }{\lim}\frac{1}{2N+1}\sum_{n=-N}^{N}\frac{1}{M}\sum_{h=0}^{M-1-\left|k\right|}\mathrm{Sigmoid}\left[x\left(h+k\right)\right]{\mathrm{Sigmoid}}^{*}\left[x\left(h\right)\right]\exp\left(jnkT^{2}\cot\rho \right)\;$$

According to the definition of the unbiasedness, we can obtain as follows:

(23) $$\mathrm{bia}\left[{\hat{\hat{R}}}_{xx,\rho }^{TS}\left(k\right)\right]=E\left[{\hat{\hat{R}}}_{xx,\rho }^{TS}\left(k\right)\right]-{\hat{R}}_{xx,\rho }^{TS}\left(k\right)\;$$

where:

(24) $$E\left[{\hat{\hat{R}}}_{xx,\rho }^{TS}\left(k\right)\right]=\frac{M-\left|k\right|}{M}{\hat{R}}_{xx,\rho }^{TS}\left(k\right)\;$$

For a given |k| value and as M→∞, we can obtain $E\left[{\hat{\hat{R}}}_{xx,\rho }^{TS}\left(k\right)\right]={\hat{R}}_{xx,\rho }^{TS}\left(k\right)$, i.e., $\mathrm{bia}\left[{\hat{\hat{R}}}_{xx,\rho }^{TS}\left(k\right)\right]=0$. Therefore, the estimator of the TS-FC is the asymptotic unbiased estimation.

According to the definition of the consistency, we can obtain:

(25) $$\begin{array}{cc} \mathrm{Var}\left[{\hat{\hat{R}}}_{xx,\rho }^{TS}\left(k\right)\right] & =E\left\{{\left[{\hat{\hat{R}}}_{xx,\rho }^{TS}\left(k\right)-E\left[{\hat{\hat{R}}}_{xx,\rho }^{TS}\left(k\right)\right]\right]}^{2}\right\}\\ & =E\left\{{\left[{\hat{\hat{R}}}_{xx,\rho }^{TS}\left(k\right)\right]}^{2}\right\}-{\left\{E\left[{\hat{\hat{R}}}_{xx,\rho }^{TS}\left(k\right)\right]\right\}}^{2} \end{array}$$

When M→∞, we can obtain $\mathrm{Var}\left[{\hat{\hat{R}}}_{xx,\rho }^{TS}\left(k\right)\right]=0$.

In summary, according to the $\mathrm{bia}\left[{\hat{\hat{R}}}_{xx,\rho }^{TS}\left(k\right)\right]=0$ and $\mathrm{Var}\left[{\hat{\hat{R}}}_{xx,\rho }^{TS}\left(k\right)\right]=0$, we may draw a conclusion that the estimator of the TS-FC is the asymptotic consistent estimation.

#### 4.3.2. Unbiasedness and Consistency of the TS-FPSD

Let the estimator of the TS-FPSD is:

(26) $${\hat{P}}_{xx,\rho }^{TS}\left(w\right)=A_{-\rho }{\tilde{F}}^{\rho }\left[{\hat{\hat{R}}}_{xx,\rho }^{TS}\left(k\right)\right]\left(w\right)\exp\left(-j\left(w^{2}/2T^{2}\right)\cot\rho \right)\;$$

According to the definition of the unbiasedness, we can obtain:

(27) $$\mathrm{bia}\left[{\hat{P}}_{xx,\rho }^{TS}\left(w\right)\right]=E\left[{\hat{P}}_{xx,\rho }^{TS}\left(w\right)\right]-P_{xx,\rho }^{TS}\left(w\right)\;$$

For a given |k| value and as M→∞,$E\left[{\hat{P}}_{xx,\rho }^{TS}\left(w\right)\right]=P_{xx,\rho }^{TS}\left(w\right)$, i.e., $\mathrm{bia}\left[{\hat{P}}_{xx,\rho }^{TS}\left(w\right)\right]=0$. Therefore, the estimator of the TS-FPSD is the asymptotic unbiased estimation.

According to the definition of the consistency, we can obtain:

(28) $$\begin{array}{cc} \mathrm{Var}\left[{\hat{P}}_{xx,\rho }^{TS}\left(w\right)\right] & =E\left\{{\left[{\hat{P}}_{xx,\rho }^{TS}\left(w\right)-E\left[{\hat{P}}_{xx,\rho }^{TS}\left(w\right)\right]\right]}^{2}\right\}\\ & =E\left\{{\left[{\hat{P}}_{xx,\rho }^{TS}\left(w\right)\right]}^{2}\right\}-{\left\{E\left[{\hat{P}}_{xx,\rho }^{TS}\left(w\right)\right]\right\}}^{2} \end{array}$$

For a given |k| and as M→∞, we can get:

(29) $$\mathrm{Var}\left[{\hat{P}}_{xx,\rho }^{TS}\left(w\right)\right]={\left[P_{xx,\rho }^{TS}\left(w\right)\right]}^{2}-{\left[E\left\{{\hat{P}}_{xx,\rho }^{TS}\left(w\right)\right\}\right]}^{2}=0\;$$

In summary, according to $\mathrm{bia}\left[{\hat{P}}_{xx,\rho }^{TS}\left(w\right)\right]=0$ and $\mathrm{Var}\left[{\hat{P}}_{xx,\rho }^{TS}\left(w\right)\right]=0$, we may draw a conclusion that the estimator of the TS-FPSD is the asymptotic consistent estimation.

## 5. Parameter Estimation Based on TS-FPSD

### 5.1. Joint Doppler Stretch and Time Delay Estimation

The echo signal y(t) with α-stable distribution noise can be expressed as:

(30) $$y\left(t\right)=\sum_{l=1}^{L}\beta_{l}x\left(\sigma_{l}\left(t-\tau_{l}\right)\right)+n\left(t\right)\;$$

where the noise n(t) denotes the SαS noise. 

According to the definition of the TS-FC, the TS-FC ${\hat{R}}_{yy,\rho }^{TS}\left(\xi \right)$ of the echo signal y(t) can be expressed as:

(31) $${\hat{R}}_{yy,\rho }^{TS}\left(\xi \right)=\underset{T\to \infty }{\lim}\frac{1}{2T}\int_{-T}^{+T}R_{yy}^{\mathrm{Sigmoid}}\left(t+\xi ,t\right)\exp\left(jt\xi \cot\rho \right)dt\;$$

According to the definition of the TS-FPSD, the TS-FPSD $P_{yy,\rho }^{TS}\left(m\right)$ of the signal y(t) can be expressed as

(32) $$P_{yy,\rho }^{TS}\left(m\right)=A_{-\rho }F^{\rho }\left[{\hat{R}}_{yy,\rho }^{TS}\left(\xi \right)\right]\left(m\right)\exp\left(-jm^{2}\cot\rho /2\right)\;$$

The joint estimation method for the Doppler stretch $\sigma_{l}$ and the time delay $\tau_{l}$ based on the TS-FPSD, is given by [16]:

(33) $$\begin{array}{l} {\hat{\sigma }}_{l}=\sqrt{-\frac{\cot\rho_{l}}{2\pi \mu_{0}}}\\ {\hat{\tau }}_{l}=\frac{2\pi f_{0}{\hat{\sigma }}_{l}-m_{l}\csc\rho_{l}}{2\pi \mu_{0}{\hat{\sigma }}_{l}^{2}}\\ \left(\rho_{l},m_{l}\right)=\underset{\rho ,m}{\mathrm{argmax}}\left|P_{yy,\rho }^{TS}\left(m\right)\right| \end{array}\}\;$$

Accordingly, the estimation of the Doppler stretch and the time delay in wideband echoes for a LFM pulse radar under an alpha-stable distribution noise was achieved via the proposed tuneable Sigmoid fractional power spectrum density function. The steps involved in this process are as follows:

**Step** **1** Obtain the echo signal y(t).
**Step** **2** Compute the TS-FC ${\hat{R}}_{yy,\rho }^{TS}\left(\xi \right)$ from Equation (31).
**Step** **3** Compute the TS-FPSD $P_{yy,\rho }^{TS}\left(m\right)$ from Equation (32).
**Step** **4** Search for the peaks of $P_{yy,\rho }^{TS}\left(m\right)$ and obtain the locations of these peaks $\left({\hat{\tau }}_{l},{\hat{\sigma }}_{l}\right)$, for l=1,…,L.
**Step** **5** **Step 5** Estimate the Doppler stretch and time delay according to Equation (33).

### 5.2. The Boundness of the TS-FPSD to the SαS Noise

We consider z(t) as an observed signal, defined as:

(34) z(t)=s(t)+n(t) 

where s(t) denotes the signal, and the noise n(t) is a sequence of the i.i.d isotropic complex SαS random variable.

According to the definition of the TS-FPSD, we can obtain:

(35) $$\begin{array}{cc} P_{zz,\rho }^{TS}\left(m\right) & =A_{-\rho }F^{\rho }\left[{\hat{R}}_{zz,\rho }^{TS}\left(\xi \right)\right]\left(m\right)\exp\left(-\frac{1}{2}jm^{2}\cot\rho \right)\\ & =A_{-\rho }\left\{A_{\rho }\int_{-\infty }^{+\infty }{\hat{R}}_{zz,\rho }^{TS}\left(\xi \right)\exp\left(j\frac{\left(m^{2}+\xi^{2}\right)\cot\rho -2m\xi \csc\rho }{2}\right)d\xi \right\}\\ & \cdot \exp\left(-\frac{jm^{2}\cot\rho }{2}\right)\\ & =A_{\rho }A_{-\rho }\int_{-\infty }^{+\infty }\exp\left(\frac{1}{2}j\xi^{2}\cot\rho -jm\xi \csc\rho \right){\hat{R}}_{zz,\rho }^{TS}\left(\xi \right)d\xi \end{array}$$

Let $t=t_{1}+\xi$,

(36) $$\begin{array}{cc} P_{zz,\rho }^{TS}\left(m\right) & =A_{\rho }A_{-\rho }\int_{-\infty }^{+\infty }\exp\left(\frac{1}{2}j\xi^{2}\cot\rho -jm\xi \csc\rho \right)d\xi \\ & \cdot \left\{\underset{T\to \infty }{\lim}\frac{1}{2T}\int_{-T}^{+T}R_{zz}^{\mathrm{Sigmoid}}\left(t_{1}+\xi ,t_{1}\right)\exp\left(jt_{1}\xi \cot\rho \right)dt_{1}\right\}\\ & =A_{\rho }A_{-\rho }\underset{T\to \infty }{\lim}\frac{1}{2T}\int_{-\infty }^{+\infty }\exp\left(\frac{1}{2}j\xi^{2}\cot\rho -jm\xi \csc\rho +jt_{1}\xi \cot\rho \right)d\xi \\ & \cdot \int_{-T}^{+T}E\left\{\mathrm{Sigmoid}\left[z(t_{1}+\xi )\right]{\mathrm{Sigmoid}}^{*}[z(t_{1})]\right\}dt_{1} \end{array}$$

According to the properties of the tuneable Sigmoid transform, the SαS process with a=0 can be transformed to a second-order moment process by the Sigmoid transform. Therefore, $E\left\{\mathrm{Sigmoid}\left[z(t_{1}+\tau )\right]{\mathrm{Sigmoid}}^{*}[z(t_{1})]\right\}$ is bounded for the SαS process because it is only involved with Sigmoid[z(t)], which can guarantees the boundness of $P_{zz,\rho }^{TS}\left(m\right)$ under the SαS noise. Furthermore, the transformation does not change the estimation result of the time delay and the Doppler frequency. Therefore, the TS-FPSD method can be used to estimate the parameters of the wideband echoes y(t) under the α-stable distribution noise.

### 5.3. Parameter Selection of the TS-FPSD

**Figure 2:**
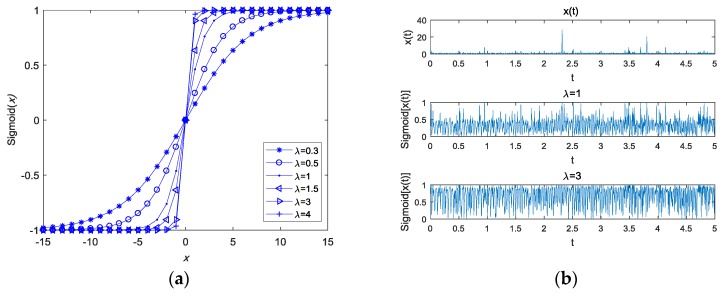
The tuneable Sigmoid function curves of the real signal with respect to λ. (**a**) x(t) is the real signal; (**b**) x(t) is the real signal with impulsive noise.

The inclined coefficient λ was used as a scale factor to fit various signals and noises. A proper λ will retain sufficient information that is associated with the time delay and Doppler stretch, and it will attenuate most of the impulsive noise at the same time. Thus, the proper selection of λ will ensure the accuracy of the estimation. According to Yu et al., the attenuation upon x(t) in the tuneable Sigmoid function changes as |x(t)| changes [26]. This concept is illustrated in Figure 2. When the signal x(t) is the real signal without noise, no matter what the value of the inclined coefficient λ is, |Sigmoid[x(t)]|<1 is true, as illustrated in Figure 2a. Furthermore, when the real signal x(t) contains the real impulsive noise, no matter what the value of the inclined coefficient λ is, |Sigmoid[x(t)]|<1 is also true, as illustrated is Figure 2b. 

**Figure 3:**
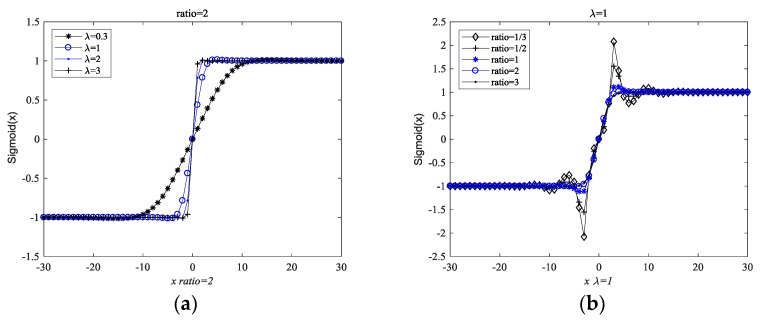
The Sigmoid function curves of the complex signal. (**a**) The Sigmoid function curves of the complex signal with respect to λ; (**b**) The Sigmoid function curves of the complex signal with respect to the ratio between its real and imaginary components.

However, when the signal x(t) is a complex signal, the above results do not always hold. Figure 3 demonstrates the Sigmoid function curves of the complex signal with respect to λ, and the ratio between the real and imaginary component. Figure 3a shows that the tuneable Sigmoid function changes with respect to λ when the ratio between the real and imaginary components is 2. Figure 3b shows that the tuneable Sigmoid function changes with respect to x(t), with the same amplitude, but with a different ratio between the real and imaginary component when the inclined coefficient λ is 1. We found that the suppression capability may be increased by increasing the ratio between the real and imaginary components. 

We observed that |Sigmoid[x(t)]|<1 may not be true when x(t) is a complex signal, as illustrated in Figure 3. Furthermore, we also found that the amplitude of the complex signal, the inclined coefficient λ, and the ratio between the real and imaginary components had some effects in suppressing the impulsive noise capability of the tuneable Sigmoid function.

**Figure 4:**
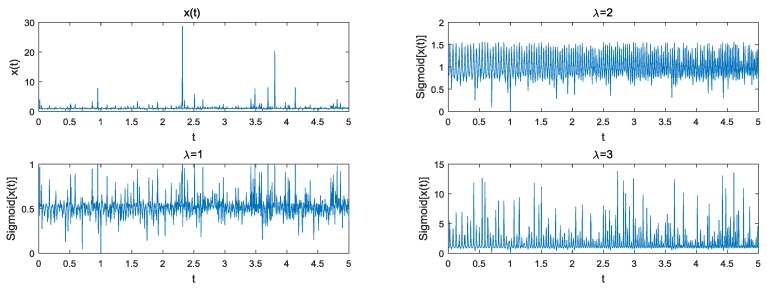
SαS noise suppression with GSNR=5dB and α=1.2 versus the inclined coefficient λ.

Figure 4 shows that the suppression ability of the tuneable Sigmoid function for a SαS noise with GSNR=5dB and α=1.2. In this simulation, the signal x(t) is the complex signal with impulsive noise. The Sigmoid function employs the tuneable parameter λ, which could be used to control the inclination of the curve. The outliers can be suppressed after the transformation. From Figure 4, we found that the suppression capability may be decreased by increasing the inclined coefficient λ, and the tuneable Sigmoid function with λ=3 fails to suppress the interference of the α-stable distribution noise. A higher inclined coefficient λ had negative impacts on the suppression capability of the impulsive noise. Therefore, the proper selection of λ affected accuracy of the estimation. 

### 5.4. Feasibility Analysis of the TS-FPSD

**Figure 5:**
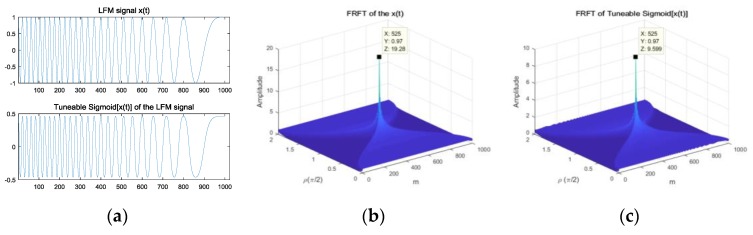
The LFM signal in the time domain and the FRFT domain. (**a**) the LFM signal and the tuneable Sigmoid[x(t)]; (**b**) the FRFT of the LFM signal; (**c**) FRFT of the the tuneable Sigmoid[x(t)].

According to Property 4 of the Sigmoid transform, the Sigmoid transform did not change the modulation characteristic of the signal, i.e., Sigmoid[x(t)] and x(t) had the same modulation characteristics. The simulation results are illustrated in Figure 5 below, to verify this property. 

**Figure 6:**
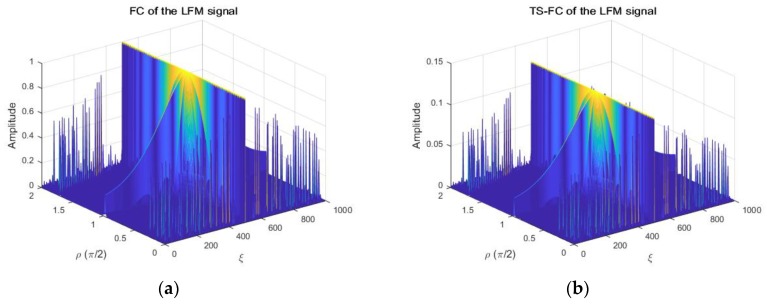

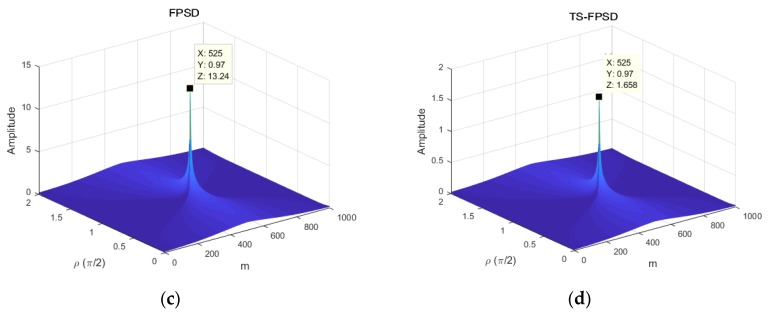
The LFM signal in the FRFT domain. (**a**) the FC of the LFM signal; (**b**) the TS-FC of the LFM signal; (**c**) the FPSD of the LFM signal; (**d**) the TS-FPSD of the LFM signal.

From Figure 5, we found that the LFM signal x(t) and the tuneable Sigmoid transform of the LFM signal Sigmoid[x(t)] had the same modulation characteristics in the time domain, and the FRFT of the LFM signal x(t) and the FRFT of the tuneable Sigmoid[x(t)] had the same peak locations in the FRFT domain. In conclusion, the tuneable Sigmoid transform did not change the modulation characteristics of the LFM signal. From Figure 6, we found that the FC of the LFM signal x(t) and the FC of the Sigmoid[x(t)] had energy concentrations at the same rotation angles in the FRFT domain. The FPSD of the LFM signal and the TS-FPSD of the LFM signal had also the same peak locations in the FRFT domain Moreover, the peak location was the same for the FRFT of the LFM signal and the FRFT of the tuneable Sigmoid[x(t)], as illustrated in Figure 5b,c and Figure 6b,c. Thus, the Sigmoid transform did not change the modulation characteristics of the signal. 

In summary, the parameters of the Doppler stretch and the time delay could be estimated by searching for the peak of the TS-FPSD.

### 5.5. The Cramer–Rao Bound

In this section, we derived a novel explicit expression for the exact Cramer–Rao Bound (CRB) on the accuracy of estimating the signal model parameters.

The CRB expresses a lower bound for the variance of an unbiased estimate and is, in general, not too difficult to compute. By comparing the performance of an estimator to the CRB, we can often have an indication on how close the estimator is to the optimum.

The echoes signal can be expressed as the following:

(37) y(t)=x(σ,τ,t)β+n(t) 

where $\beta ≜{\left[\beta_{1},\ldots ,\beta_{L}\right]}^{T}$,$x\left(a,\tau ,t\right)=\left[x\left(a_{1}\left(t-\tau_{1}\right)\right),x\left(a_{2}\left(t-\tau_{2}\right)\right),\ldots ,x\left(a_{L}\left(t-\tau_{L}\right)\right)\right]$.

The two parameters to be estimated are the time delay τ and the Doppler stretch σ, which form the parameter vector ξ, such that $\xi ={\left[\sigma ,\tau \right]}^{T}$,where ${\left[\right]}^{T}$ denotes the transpose of a vector, $\sigma ≜\left[\sigma_{1},\sigma_{2},\ldots ,\sigma_{L}\right]$, and $\tau ≜\left[\tau_{1},\tau_{2},\ldots ,\tau_{L}\right]$. Suppose that the number of snapshots is N.

First, we obtained closed-form expressions for all particular sub-blocks of the Fisher information matrix (FIM). The element i,j of the FIM for estimating the vector $\xi ={\left[\sigma ,\tau \right]}^{T}$ can be shown as [29,30,31]:

(38) FIMij=NTr(Qn−1∂Qn∂ξiQn−1∂Qn∂ξj)+2Re∑t=1N{(∂x(σ,τ)β∂ξi)HQn−1(∂x(σ,τ)β∂ξj)}

We assumed that the noise was a sequence of the i.i.d isotropic complex SαS random variable. The geometric power $S_{0}$ is used to represent the power of symmetric α-stable random noise, i.e., $Q_{n}=S_{0}I_{N}$. Since x(σ,τ,t)β and $Q_{n}$ depend on different elements of ξ, it is clear that FIM will be block diagonal with respect to the signal ($\xi ={\left[\sigma ,\tau \right]}^{T}$) and noise parameters. In particular, the first term of Equation (38) will give a nonzero result only for the noise block. Since we are concerned only with the CRB for the signal parameters, we need only consider the second term:

(39) FIMij(ξ)=2Re∑t=1N{(∂x(σ,τ,t)β∂ξi)HQn−1(∂x(σ,τ,t)β∂ξj)} 

Using Equations (38) and (39), the following explicit expressions for the blocks of the FIM are derived as follows:

(40) FIMσσ(ξ)=2Re∑t=1N{ΔH(xσ′(σ,τ,t))HQn−1(xσ′(σ,τ,t))Δ} 

(41) FIMττ(ξ)=2Re∑t=1N{ΔH(x′τ(σ,τ,t))HQn−1(x′τ(σ,τ,t))Δ} 

(42) FIMστ(ξ)=2Re∑t=1N{ΔH(x′σ(σ,τ,t))HQn−1(x′τ(σ,τ,t))Δ} 

where:

(43) $$\Delta ≜diag\left\{\beta_{1},\beta_{2},\ldots ,\beta_{L}\right\}\;$$

(44) $${x^{\prime }}_{\sigma }\left(\sigma ,\tau ,t\right)≜\frac{\partial x_{\sigma }\left(\sigma ,\tau ,t\right)}{\partial \sigma }=\left[{x^{\prime }}_{\left(\sigma \right)}\left(\sigma ,\tau ,t\right)\right]\;$$

(45) $${x^{\prime }}_{\left(\sigma \right)}\left(\sigma ,\tau ,t\right)≜\left[\frac{\partial x\left(\sigma_{1}\left(t-\tau_{1}\right)\right)}{\partial \sigma_{1}},\frac{\partial x\left(\sigma_{2}\left(t-\tau_{2}\right)\right)}{\partial \sigma_{2}},\ldots ,\frac{\partial x\left(\sigma_{L}\left(t-\tau_{L}\right)\right)}{\partial \sigma_{L}}\right]\;$$

(46) $${x^{\prime }}_{\tau }\left(\sigma ,\tau ,t\right)≜\frac{\partial x_{\tau }\left(\sigma ,\tau ,t\right)}{\partial \tau }=\left[{x^{\prime }}_{\left(\tau \right)}\left(\sigma ,\tau ,t\right)\right]\;$$

(47) $${x^{\prime }}_{\left(\tau \right)}\left(\sigma ,\tau ,t\right)≜\left[\frac{\partial x\left(\sigma_{1}\left(t-\tau_{1}\right)\right)}{\partial \tau_{1}},\frac{\partial x\left(\sigma_{2}\left(t-\tau_{2}\right)\right)}{\partial \tau_{2}},\ldots ,\frac{\partial x\left(\sigma_{L}\left(t-\tau_{L}\right)\right)}{\partial \tau_{L}}\right]\;$$

The expression for the CRB, shown in Equation (48), is obtained by substituting Equations (40)–(47) into Equation (39):

(48) $$CRB\left(\xi \right)=FIM^{-1}\;$$

## 6. Simulation Results

In this section, we performed four types of simulation experiments to evaluate the relative performances of the FPSD [12], the FLOS-FPSD [16], and the TS-FPSD methods under the α-stable distribution noise, respectively.

The parameters of the transmitted LFM signal in the simulation are assumed as follows. The initial frequency $f_{0}=0.2f_{s}$ and the modulation rate is set to $\mu_{0}=0.1f_{s}^{2}/N$. The sampling rate is set to $f_{s}=1\;\mathrm{MHz}$ with a sampling length of N=1000, *T* = 1 ms. The number of multipath is L=2, and the Doppler stretch and time delay are set to $\sigma_{1}=0.9$,$\sigma_{2}=1.1$,$\tau_{1}=20/f_{s}$ and $\tau_{2}=60/f_{s}$, respectively. The Root Mean Square Error (RMSE) is defined as:

(49) $$RMSE=\frac{1}{2}\left(\sqrt{\frac{1}{K}{\sum_{k=1}^{K}\left[{\hat{x}}_{1}\left(k\right)-x_{1}\right]}^{2}}+\sqrt{\frac{1}{K}{\sum_{k=1}^{K}\left[{\hat{x}}_{2}\left(k\right)-{\hat{x}}_{2}\right]}^{2}}\right)\;$$

where ${\hat{x}}_{1}$ and ${\hat{x}}_{2}$ are the estimation of $x_{1}$ and $x_{2}$, and K is the Monte Carlo number. The RMSE of the time delay and Doppler stretch can be expressed as:

(50) $$RMSE_{\tau }=\frac{1}{2}\left(\sqrt{\frac{1}{K}{\sum_{k=1}^{K}\left[{\hat{\tau }}_{1}\left(k\right)-\tau_{1}\right]}^{2}}+\sqrt{\frac{1}{K}{\sum_{k=1}^{K}\left[{\hat{\tau }}_{2}\left(k\right)-\tau_{2}\right]}^{2}}\right)\;$$

and:

(51) $$RMSE_{\sigma }=\frac{1}{2}\left(\sqrt{\frac{1}{K}{\sum_{k=1}^{K}\left[{\hat{\sigma }}_{1}\left(k\right)-\sigma_{1}\right]}^{2}}+\sqrt{\frac{1}{K}{\sum_{k=1}^{K}\left[{\hat{\sigma }}_{2}\left(k\right)-\sigma_{2}\right]}^{2}}\right)\;$$

The numbers of Monte Carlo runs was set to 200 in Simulations 2 and 3.

### Simulation 1: Estimation Accuracy with Respect to λ

**Figure 7:**
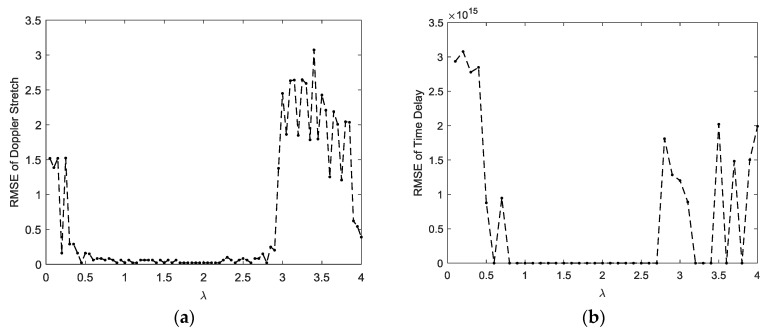
Estimation accuracy with respect to λ. (**a**) RMSE of the Doppler stretch; (**b**) RMSE of the time delay.

To evaluate the performance of the TD and DS with respect to λ in this simulation, the characteristic exponent α was set to α=1.2 and GSNR=5dB. The RMSE was used to evaluate the performance of the TS-FPSD with respect to λ, as illustrated is Figure 7. 

From Figure 7, we observed that the performance estimation of the TD and DS using λ∈[0.8,2.7] provided a better performance than that using other λ values, for the case of the alpha-stable noise. Therefore, the inclined coefficient λ for the TS-FPSD was set as λ=1 in all the later simulations of this paper. 

### Simulation 2: FPSD, FLOS-FPSD, and TS-FPSD for a Single Estimation

**Figure 8:**
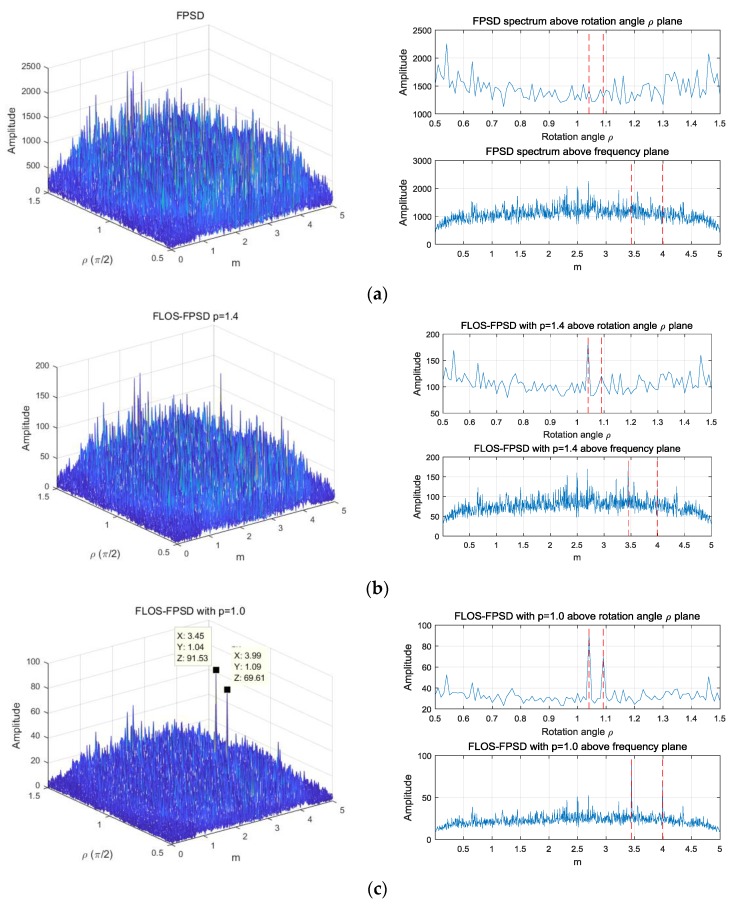

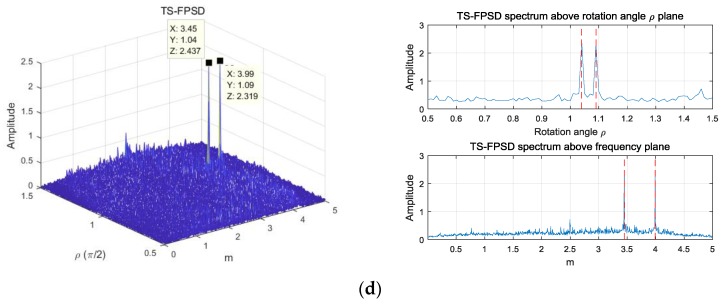
The spectrum of FPSD, FLOS-FPSD, and TS-FPSD under the SαS noise with GSNR=5dB and α=1.3. (**a**) The FPSD spectrum of the echo and its rotation angle plane and the frequency plane; (**b**) The FLOS-FPSD with a p=1.4 spectrum of the echo, and its rotation angle plane and the frequency plane; (**c**) The FLOS-FPSD with p=1.0 spectrum of the echo, and its rotation angle plane and the frequency plane; (**d**) The TS-FPSD spectrum of the echo, and its rotation angle plane and the frequency plane.

**Figure 9:**
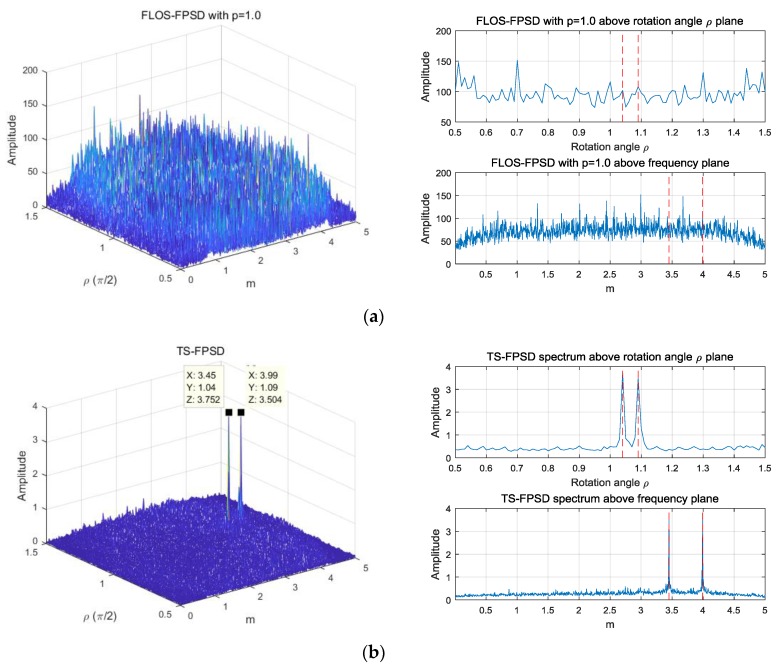
The spectrum of FPSD, FLOS-FPSD, and TS-FPSD under the SαS noise with GSNR=5 dB and α=1.1; (**a**) The FLOS-FPSD with p=1.0 spectrum of the echo, and its rotation angle plane and the frequency plane; (**b**) The TS-FPSD spectrum of the echo, and its rotation angle plane and the frequency plane.

Figure 8 and Figure 9 show the estimation results of the FPSD, FLOS-FPSD, and TS-FPSD for a single trial of data under the SαS noise with GSNR=5dB and α=1.3 and α=1.1. In order to show the peak location information and the performance of the algorithm more clearly, the 2D rotation angle plane and the 2D frequency plane are shown. The 2D rotation angle plane and 2D frequency plane could better demonstrate the peak location. In Figure 8 and Figure 9, the red line denotes the true values of the rotation angle and frequency in the FRFT domain. Futhermore, Table 1 shows the comparison of three algorithms for impulsive noise suppression.

From Figure 8a, we found that the FPSD algorithm failed when the SαS noise occurred. The reason is that the FPSD method did not have the ability to suppress impulsive noise. Since the second-order moment of a SαS random variable with 0<α<2 does not exist, and the fractional correlation function was based on second-order moments, the performance of the FPSD degraded severely. The FLOS-FPSD algorithm, combining the fractional lower order statistics theory with the fractional power spectrum density function, effectively suppressed the α-stable distribution noise interference, so the FLOS-FPSD could obtain a clear peak under the SαS noise of GSNR=5dB, with α=1.3 and p=1.0. However, the FLOS-FPSD failed to obtain the correct spectrum peak under the SαS noise with α=1.3 and p=1.4, mainly due to the fact the fractional lower-order moment p value was not appropriate, as illustrated in Figure 8b,c. In fractional lower order statistics theory, the characteristic exponent of the noise must be estimated to ensure that 1≤p<α or 0<p<α/2, otherwise the algorithm performance can degrade seriously, and may even become invalid, while the fractional lower-order moment value is not appropriate.

The FLOS-FPSD failed to obtain the correct spectrum peak under the α-stable distribution noise GSNR=5dB with α=1.1 and p=1.0; however, the TS-FPSD peak could be easily separated from the impulsive noise, as illustrated in Figure 9, mainly due to the fact that the Sigmoid transform could suppress the impulsive noise better than the FLOS. Therefore, the performance of the TS-FPSD outweighed those of the FLOS-FPSD. The proposed method based on the TS-FFPSD could effectively suppress impulsive noise interference, yielded an accurate peak estimation, and had a better estimation performance.

### Simulation 3: Estimation Accuracy with Respect to GSNR

**Figure 10:**
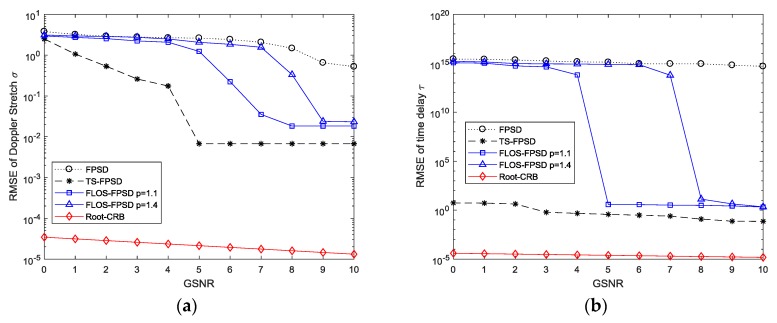
Estimation accuracy versus the generalized signal-noise-ratio (GSNR). (**a**) RMSE of the Doppler stretch; (**b**) RMSE of the time delay.

To evaluate the performance of the time delay (TD) and the Doppler stretch (DS) in this simulation, the characteristic exponent α was set to α=1.2 and the fractional lower order moment p was set to p=1.1 and p=1.4 for the FLOS-FPSD method, respectively. The resulting RMSE performance versus GSNR is illustrated in Figure 10. 

From Figure 10, we can find that the FPSD method had a poor estimation performance with the SαS noise interference. On the other hand, combining the fractional lower-order statistics theory with the fractional power spectrum density, the FLOS-FPSD method with α=1.2 and p=1.1 could effectively suppress the SαS noise interference. Accordingly, the FLOS-FPSD method yielded a clear peak under the SαS noise. However, the performance was affected by the fractional lower-order moment p value. The FLOS-FPSD method, with α=1.2 and p=1.4 could not accurately estimate the parameters, because the fractional lower-order moment value was not appropriate. On the contrary, the TS-FPSD method could not suppress the SαS noise interference, employing the tuneable Sigmoid transform, but the estimation performance of the TS-FPSD also could not be affected by the fractional lower-order moment p value. Therefore, the performance of the TS-FPSD method outweighed those of the FLOS-based method.

### Simulation 4: Estimation Accuracy with Respect to the Characteristic Exponent α

**Figure 11:**
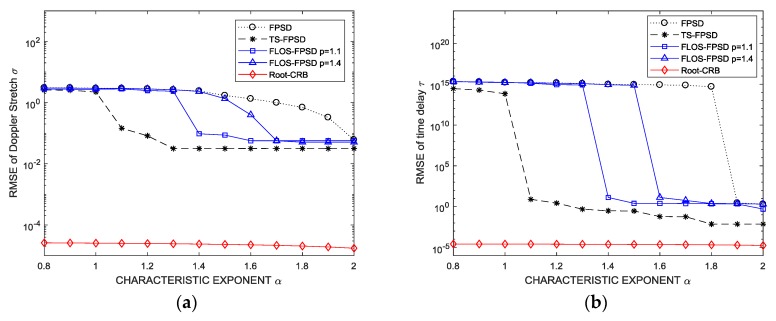
Estimation accuracy versus the characteristic exponent. (**a**) RMSE of the Doppler stretch; (**b**) RMSE of the time delay.

In this simulation, the GSNR was set to 5 dB, and the fractional lower-order moment p was set to p=1.1 and p=1.4 for the FLOS-FPSD method, respectively. Figure 11 shows the performance versus the characteristic exponent α. From Figure 11, we found that the FPSD algorithm had a better estimation performance when the characteristic exponent α was close to 2. The FLOS-FPSD method may suppress the α-stable distribution noise interference by employing the fractional lower-order statistic theory. The performance of the FLOS-FPSD method was shown to be better than that of the FPSD method. 

Since the FLOS and the Sigmoid transform methods could both suppress the impulsive noise, the suppression capacity of the FLOS method was not sufficient, and the Sigmoid function suppressed the outliers much harder than did the FLOS. Therefore, the estimation performance of the TS-FPSD algorithm was superior to that of FLOS-FPSD algorithm. 

## 7. Conclusions

In this paper, two novel concepts, the tuneable Sigmoid transform fractional correlation function and the tuneable Sigmoid transform fractional power spectrum density function, are proposed to estimate the time delay and Doppler stretch of the wideband echoes of a LFM pulse radar signal under the presence of an impulsive noise environment. Then, the unbiased estimation and consistent estimation of the algorithm are derived. Furthermore, the boundness of the TS-FPSD to the SαS noise, the parameter selection of the TS-FPSD, the feasibility analysis of the TS-FPSD, and the Cramér–Rao bound for parameter estimation are presented, to evaluate the performance of the proposed method. The simulation results and theoretical analysis are presented to illustrate the validity of the foregoing method. It is clearly shown that the proposed method cannot only can effectively restrain impulsive noise interference, but it also does not depend on a priori knowledge of the noise. In addition, it yields a higher estimation accuracy and a lower computational complexity in the impulsive noise environment.

## Figures and Tables

**Table 1:** A comparison of the three algorithms for impulsive noise suppression.

Algorithms	Impulsive Noise Suppression	Obtaining the Correct Spectrum Peak	Performance of the Parameter Estimation
FPSD	Worse	Cannot	Poor
FLOS-FPSD with inappropriate p	Worse	Occasionally	Poor
FLOS-FPSD with appropriate p	Average	Sometimes	Average
TS-FPSD	Better	Always	Better

## Appendix A

In this section, the derivation details of the Equations (10)–(12) is presented.

Let $y\left(t\right)=y_{1}\left(t\right)+\ldots +y_{l}\left(t\right)+\ldots +y_{L}\left(t\right)+n\left(t\right)$, where $y_{l}\left(t\right)=\beta_{l}x\left(\sigma_{l}\left(t-\tau_{l}\right)\right)+n_{l}\left(t\right)$.

From Equations (2) and (6), the fractional correlation ${\hat{R}}_{yy}^{\rho }\left(\xi \right)$ of the echo signal y(t) can be expressed as:

(A1) $$\begin{array}{cc} {\hat{R}}_{yy}^{\rho }\left(\xi \right) & =\underset{T\to \infty }{\lim}\frac{1}{2T}\int_{-T}^{+T}R_{yy}\left(t+\xi \right)\exp\left(jt\xi \cot\rho \right)dt\\ & =\underset{T\to \infty }{\lim}\frac{1}{2T}\int_{-T}^{+T}E\left\{\left[y_{1}\left(t+\xi \right)+\ldots +y_{L}\left(t+\xi \right)+n\left(t+\xi \right)\right]{\left[y_{1}\left(t\right)+\ldots +y_{L}\left(t\right)+n\left(t\right)\right]}^{*}\right\}\exp\left(jt\xi \cot\rho \right)dt\\ & =\underset{T\to \infty }{\lim}\frac{1}{2T}\int_{-T}^{+T}E\{[y_{1}\left(t+\xi \right)y_{1}^{*}\left(t\right)+\ldots +y_{L}\left(t+\xi \right)y_{L}^{*}\left(t\right)+y_{1}\left(t+\xi \right)y_{2}^{*}\left(t\right)+\ldots \\ & +y_{L}\left(t+\xi \right)y_{L-1}^{*}\left(t\right)+n\left(t+\xi \right)n^{*}\left(t\right)]\}\exp\left(jt\xi \cot\rho \right)dt\\ & =\underset{T\to \infty }{\lim}\frac{1}{2T}\int_{-T}^{+T}\left\{E\left[y_{1}\left(t+\xi \right)y_{1}^{*}\left(t\right)+\ldots +y_{L}\left(t+\xi \right)y_{L}^{*}\left(t\right)\right]\right\}\exp\left(jt\xi \cot\rho \right)dt\\ & +\underset{T\to \infty }{\lim}\frac{1}{2T}\int_{-T}^{+T}\left\{E\left[y_{1}\left(t+\xi \right)y_{2}^{*}\left(t\right)+\ldots +y_{l}\left(t+\xi \right)y_{q}^{*}\left(t\right)+\ldots +y_{L}\left(t+\xi \right)y_{L-1}^{*}\left(t\right)\right]\right\}\exp\left(jt\xi \cot\rho \right)dt\\ & +\underset{T\to \infty }{\lim}\frac{1}{2T}\int_{-T}^{+T}\left\{E\left[y\left(t+\xi \right)n^{*}\left(t\right)+n\left(t+\xi \right)y^{*}\left(t\right)\right]\right\}\exp\left(jt\xi \cot\rho \right)dt\\ & +\underset{T\to \infty }{\lim}\frac{1}{2T}\int_{-T}^{+T}\left\{E\left[n\left(t+\xi \right)n^{*}\left(t\right)\right]\right\}\exp\left(jt\xi \cot\rho \right)dt \end{array}$$

Let:

(A2) $$\begin{array}{cc} {\hat{R}}_{yy,l}^{\rho }\left(\xi \right) & =\underset{T\to \infty }{\lim}\frac{1}{2T}\int_{-T}^{+T}\left\{E\left[y_{1}\left(t+\xi \right)y_{1}^{*}\left(t\right)+\ldots +y_{L}\left(t+\xi \right)y_{L}^{*}\left(t\right)\right]\right\}exp\left(jt\xi \cot\rho \right)dt\\ & =\underset{T\to \infty }{\lim}\frac{1}{2T}\int_{-T}^{+T}\sum_{l=1}^{L}E\left[y_{l}\left(t+\xi \right)y_{l}^{*}\left(t\right)\right]exp\left(jt\xi \cot\rho \right)dt \end{array}$$

(A3) $$\begin{array}{cc} {\hat{R}}_{yy,lq}^{\rho }\left(\xi \right) & =\underset{T\to \infty }{\lim}\frac{1}{2T}\int_{-T}^{+T}\left\{E\left[y_{1}\left(t+\xi \right)y_{2}^{*}\left(t\right)+\ldots +y_{l}\left(t+\xi \right)y_{q}^{*}\left(t\right)+\ldots +y_{L}\left(t+\xi \right)y_{L-1}^{*}\left(t\right)\right]\right\}exp\left(jt\xi \cot\rho \right)dt\\ & =\underset{T\to \infty }{\lim}\frac{1}{2T}\int_{-T}^{+T}\sum_{l\neq q}^{L}E\left[y_{l}\left(t+\xi \right)y_{q}^{*}\left(t\right)\right]exp\left(jt\xi \cot\rho \right)dt \end{array}$$

(A4) $${\hat{R}}_{yn}^{\rho }\left(\xi \right)=\underset{T\to \infty }{\lim}\frac{1}{2T}\int_{-T}^{+T}\left\{E\left[y\left(t+\xi \right)n^{*}\left(t\right)+n\left(t+\xi \right)y^{*}\left(t\right)\right]\right\}exp\left(jt\xi \cot\rho \right)dt\;$$

(A5) $${\hat{R}}_{nn}^{\rho }\left(\xi \right)=\underset{T\to \infty }{\lim}\frac{1}{2T}\int_{-T}^{+T}\left\{E\left[n\left(t+\xi \right)n^{*}\left(t\right)\right]\right\}exp\left(jt\xi \cot\rho \right)dt\;$$

where ${\hat{R}}_{yn}^{\rho }\left(\xi \right)$ denotes the fractional correlation functions between the echo signal y(t) and the noise n(t), and ${\hat{R}}_{yn}^{\rho }\left(\xi \right)$ is treated as a random interference. ${\hat{R}}_{nn}^{\rho }\left(\xi \right)$ denotes the fractional correlation functions of the noise n(t).

Substituting Equations (A2)–(A5) into Equation (A1), we can rewrite Equation (A1) as follows:

(A6) $${\hat{R}}_{yy}^{\rho }\left(\xi \right)={\hat{R}}_{yy,l}^{\rho }\left(\xi \right)+{\hat{R}}_{yy,l}^{\rho }\left(\xi \right)+{\hat{R}}_{yn}^{\rho }\left(\xi \right)+{\hat{R}}_{nn}^{\rho }\left(\xi \right)\;$$

According to $y_{l}\left(t\right)$ and Equation (A2), we can obtain:

(A7) $$\begin{array}{cc} {\hat{R}}_{yy,l}^{\rho }\left(\xi \right) & =\sum_{l=1}^{L}\underset{T\to \infty }{\lim}\frac{1}{2T}\int_{-T}^{+T}E\left[y_{l}\left(t+\xi \right)y_{l}^{*}\left(t\right)\right]\exp\left(jt\xi \cot\rho \right)dt\\ & =\sum_{l=1}^{L}\underset{T\to \infty }{\lim}\frac{1}{2T}\int_{-T}^{+T}E\left\{\beta_{l}^{2}A^{2}\exp\left(j2\text{π}\left(f_{0}\sigma_{l}-\mu_{0}\tau_{l}\sigma_{l}^{2}\right)\xi +\frac{1}{2}\mu_{0}\sigma_{l}^{2}\xi^{2}+2\text{π}\mu_{0}\sigma_{l}^{2}t\xi \right)\right\}\exp\left(jt\xi \cot\rho \right)dt\\ & =\sum_{l=1}^{L}\beta_{l}^{2}A^{2}\exp\left(j2\text{π}\left(f_{0}\sigma_{l}-\mu_{0}\tau_{l}\sigma_{l}^{2}\right)\xi +\frac{1}{2}\mu_{0}\sigma_{l}^{2}\xi^{2}\right)\underset{T\to \infty }{\lim}\frac{1}{2T}\int_{-T}^{+T}dt\int_{-T}^{+T}\exp\left(j\left(2\text{π}\mu_{0}\sigma_{l}^{2}+\cot\rho \right)t\xi \right)dt \end{array}$$

When $2\pi \mu_{0}\sigma_{l}^{2}=-\cot\rho_{l}$, Equation (A7) can be rewritten as:

(A8) $${\hat{R}}_{yy,l}^{\rho_{l}}\left(\xi \right)=\sum_{l=1}^{L}\beta_{l}^{2}A^{2}\exp\left(j2\pi \left(f_{0}\sigma_{l}-\mu_{0}\tau_{l}\sigma_{l}^{2}\right)\xi +\frac{1}{2}\mu_{0}\sigma_{l}^{2}\xi^{2}\right)\;$$

From Equation (A8), we can find that ${\hat{R}}_{yy,l}^{\rho_{l}}\left(\xi \right)$ has the expression of a linear frequency modulation signal on delay ξ. The value ${\hat{R}}_{yy,l}^{\rho_{l}}\left(\xi \right)$ has the characteristic of energy that is concentrated in the FRFT domain.

(A9) $$\begin{array}{cc} {\hat{R}}_{yy,lq}^{\rho }\left(\xi \right) & =\underset{T\to \infty }{\lim}\frac{1}{2T}\sum_{l\neq q}^{L}\beta_{l}\beta_{q}A^{2}\exp\left(j2\pi \left(\frac{1}{2}\mu_{0}\left(\sigma_{l}^{2}\tau_{l}^{2}-\sigma_{q}^{2}\tau_{q}^{2}+\sigma_{l}^{2}\xi^{2}-2\sigma_{l}^{2}\tau_{l}\xi \right)-f_{0}\left(\sigma_{l}\tau_{l}-\sigma_{q}\tau_{q}-\tau_{l}\xi \right)\right)\right)\\ & \cdot \int_{-T}^{+T}\exp\left(j\left(2\pi f_{0}\left(\sigma_{l}-\sigma_{q}\right)-\pi \mu_{0}\left(\sigma_{l}^{2}\tau_{l}-\sigma_{q}^{2}\tau_{q}-2\sigma_{l}^{2}\xi \right)+\xi \cot\rho \right)t+\pi \mu_{0}\left(\sigma_{l}^{2}-\sigma_{q}^{2}\right)t^{2}\right)dt \end{array}$$

However, ${\hat{R}}_{yy,lq}^{\rho_{l}}\left(\xi \right)$, ${\hat{R}}_{yn}^{\rho_{l}}\left(\xi \right)$ and ${\hat{R}}_{nn}^{\rho_{l}}\left(\xi \right)$ do not have the characteristics of energy that is concentrated in the FRFT domain, the amplitudes of the TS-FPSD of different targets are very low at $\left(\rho_{l},m_{l}\right)$, and these signals are not considered as random interference. So we can obtain:

(A10) $${\hat{R}}_{yy}^{\rho_{l}}\left(\xi \right)=\sum_{l=1}^{L}\beta_{l}^{2}A^{2}\exp\left(j2\pi \left(f_{0}\sigma_{l}-\mu_{0}\tau_{l}\sigma_{l}^{2}\right)\xi +\frac{1}{2}\mu_{0}\sigma_{l}^{2}\xi^{2}\right)+{\hat{R}}_{yy,lq}^{\rho_{l}}\left(\xi \right)+{\hat{R}}_{yn}^{\rho_{l}}\left(\xi \right)+{\hat{R}}_{nn}^{\rho_{l}}\left(\xi \right)\;$$

Therefore, the fractional power spectrum density $P_{yy}^{\rho }\left(m\right)$ of the echo signal y(t) can be computed as:

(A11) $$\begin{array}{cc} P_{yy}^{\rho }\left(m\right) & =A_{-\rho }F^{\rho }\left[{\hat{R}}_{yy}^{\rho_{l}}\left(\tau \right)\right]\left(m\right)\exp\left(-jm^{2}\cot\rho /2\right)\\ & =P_{yy,l}^{\rho }\left(m\right)+P_{yy,lq}^{\rho }\left(m\right)+P_{yn}^{\rho }\left(m\right)+P_{nn}^{\rho }\left(m\right) \end{array}$$

where $P_{yy,lq}^{\rho }\left(m\right)$, $P_{yn}^{\rho }\left(m\right)$ and $P_{nn}^{\rho }\left(m\right)$ are not considered as random interference. Let $P_{N}^{\rho }\left(m\right)=P_{yy,lq}^{\rho }\left(m\right)+P_{yn}^{\rho }\left(m\right)+P_{nn}^{\rho }\left(m\right)$, the fractional power spectrum density $P_{yy}^{\rho }\left(m\right)$ of the y(t) can be rewritten as:

(A12) $$\begin{array}{cc} P_{yy}^{\rho }\left(m\right) & =A_{-\rho }F^{\rho }\left[{\hat{R}}_{yy}^{\rho_{l}}\left(\tau \right)\right]\left(m\right)\exp\left(-jm^{2}\cot\rho /2\right)\\ & =\sum_{l=1}^{L}A_{-\rho }A_{\rho }\int_{-\frac{T}{2}}^{+\frac{T}{2}}\exp\left(j\left(2\pi f_{0}\sigma_{l}-2\pi \mu_{0}\tau_{l}\sigma_{l}^{2}-m\csc\rho \right)\xi +\frac{j}{2}\left(2\pi \mu_{0}\sigma_{l}^{2}+\cot\rho \right)\xi^{2}\right)d\xi \\ & +P_{N}^{\rho }\left(m\right) \end{array}$$

## Appendix B

In this section, the unbiasedness and consistency are derived.

The definitions of the unbiasedness and consistency can be shown as:

If the following holds:

(A13) $$\mathrm{bia}\left[\hat{\theta }\right]=E\left[\hat{\theta }\right]-\theta =0\;$$

Then, $\hat{\theta }$ is an unbiased estimator of the parameter θ. Otherwise, $\hat{\theta }$ is a biased estimator of θ.

and if the following holds:

(A14) $$\left\{ \begin{array}{l} \mathrm{bia}\left[\hat{\theta }\right]=E\left[\hat{\theta }\right]-\theta =0\\ Var\left[\hat{\theta }\right]=E{\left\{\hat{\theta }-E\left[\hat{\theta }\right]\right\}}^{2}=0 \end{array}\right.\;$$

Then,$\hat{\theta }$ is a consistent estimator of the parameter θ. Otherwise, $\hat{\theta }$ is not a consistent estimator of the parameter θ.

### Appendix B.1. Unbiasedness and Consistency of the TS-FC

Let the estimator of the TS-FC ${\hat{\hat{R}}}_{xx,\rho }^{TS}\left(k\right)$ be:

(A15) $${\hat{\hat{R}}}_{xx,\rho }^{TS}\left(k\right)=\underset{N\to \infty }{\lim}\frac{1}{2N+1}\sum_{n=-N}^{N}\frac{1}{M}\sum_{h=0}^{M-1-\left|k\right|}\mathrm{Sigmoid}\left[x\left(h+k\right)\right]{\mathrm{Sigmoid}}^{*}\left[x\left(h\right)\right]\exp\left(jnkT^{2}\cot\rho \right)\;$$

According to the definition of the unbiasedness, we can obtain as follows:

(A16) $$\mathrm{bia}\left[{\hat{\hat{R}}}_{xx,\rho }^{TS}\left(k\right)\right]=E\left[{\hat{\hat{R}}}_{xx,\rho }^{TS}\left(k\right)\right]-{\hat{R}}_{xx,\rho }^{TS}\left(k\right)\;$$

Where:

(A17) $$\begin{array}{cc} E\left[{\hat{\hat{R}}}_{xx,\rho }^{TS}\left(k\right)\right] & =E\left[\underset{N\to \infty }{\lim}\frac{1}{2N+1}\sum_{n=-N}^{N}\exp\left(jnkT^{2}\cot\rho \right)\frac{1}{M}\sum_{h=0}^{M-1-\left|k\right|}\mathrm{Sigmoid}\left[x\left(h+k\right)\right]{\mathrm{Sigmoid}}^{*}\left[x\left(h\right)\right]\right]\\ & =\underset{N\to \infty }{\lim}\frac{1}{2N+1}\sum_{n=-N}^{N}\exp\left(jnkT^{2}\cot\rho \right)\frac{1}{M}\sum_{h=0}^{M-1-\left|k\right|}E\left[\mathrm{Sigmoid}\left[x\left(h+k\right)\right]{\mathrm{Sigmoid}}^{*}\left[x\left(h\right)\right]\right]\\ & =\frac{M-\left|k\right|}{M}{\hat{R}}_{xx,\rho }^{TS}\left(k\right) \end{array}$$

When |k| is given and M→∞, we can obtain $E\left[{\hat{\hat{R}}}_{xx,\rho }^{TS}\left(k\right)\right]={\hat{R}}_{xx,\rho }^{TS}\left(k\right)$, i.e., $\mathrm{bia}\left[{\hat{\hat{R}}}_{xx,\rho }^{TS}\left(k\right)\right]=0$. Therefore the estimator of the TS-FC is the asymptotic unbiased estimation.

According to the definition of the consistency, we can obtain as follows:

(A18) $$\begin{array}{cc} \mathrm{Var}\left[{\hat{\hat{R}}}_{xx,\rho }^{TS}\left(k\right)\right] & =E\left\{{\left[{\hat{\hat{R}}}_{xx,\rho }^{TS}\left(k\right)-E\left[{\hat{\hat{R}}}_{xx,\rho }^{TS}\left(k\right)\right]\right]}^{2}\right\}\\ & =E\left\{{\left[{\hat{\hat{R}}}_{xx,\rho }^{TS}\left(k\right)\right]}^{2}\right\}-{\left\{E\left[{\hat{\hat{R}}}_{xx,\rho }^{TS}\left(k\right)\right]\right\}}^{2} \end{array}$$

where:

(A19) $${\left\{E\left[{\hat{\hat{R}}}_{xx,\rho }^{TS}\left(k\right)\right]\right\}}^{2}={\left[\frac{M-\left|k\right|}{M}{\hat{R}}_{xx,\rho }^{TS}\left(k\right)\right]}^{2}\;$$

(A20) $$\begin{array}{cc} E\left\{{\left[{\hat{\hat{R}}}_{xx,\rho }^{TS}\left(k\right)\right]}^{2}\right\} & =E\{\left(\underset{N\to \infty }{\lim}\frac{1}{2N+1}\sum_{n=-N}^{N}\exp\left(jnkT^{2}\cot\rho \right)\frac{1}{M}\sum_{h=0}^{M-1-\left|k\right|}\text{Sigmoid}\left[x\left(h+k\right)\right]{\text{Sigmoid}}^{*}\left[x\left(h\right)\right]\right)\\ & \cdot \left(\underset{N\to \infty }{\lim}\frac{1}{2N+1}\sum_{n=-N}^{N}\exp\left(jnkT^{2}\cot\rho \right)\frac{1}{M}\sum_{p=0}^{M-1-\left|k\right|}\text{Sigmoid}\left[x\left(p+k\right)\right]{\text{Sigmoid}}^{*}\left[x\left(p\right)\right]\right)\}\\ & =\underset{N\to \infty }{\lim}\frac{1}{2N+1}\sum_{n=-N}^{N}\exp\left(jnkT^{2}\cot\rho \right)\underset{N\to \infty }{\lim}\frac{1}{2N+1}\sum_{n=-N}^{N}\exp\left(jnkT^{2}\cot\rho \right)\\ & \cdot \left\{\frac{1}{M^{2}}\sum_{h=0}^{M-1-\left|k\right|}\sum_{p=0}^{M-1-\left|k\right|}E\left[{\text{Sigmoid}}^{*}\left[x\left(h\right)\right]{\text{Sigmoid}}^{*}\left[x\left(p\right)\right]\text{Sigmoid}\left[x\left(h+k\right)\right]\text{Sigmoid}\left[x\left(p+k\right)\right]\right]\right\}\\ & =\underset{N\to \infty }{\lim}\frac{1}{2N+1}\sum_{n=-N}^{N}\exp\left(jnkT^{2}\cot\rho \right)\underset{N\to \infty }{\lim}\frac{1}{2N+1}\sum_{n=-N}^{N}\exp\left(jnkT^{2}\cot\rho \right)\\ & \cdot \left\{\frac{1}{M^{2}}\sum_{h=0}^{M-1-\left|k\right|}\sum_{p=0}^{M-1-\left|k\right|}\left\{{\left[R_{xx}^{\text{Sigmoid}}\left(h-p\right)\right]}^{2}+{\left[R_{xx}^{\text{Sigmoid}}\left(k\right)\right]}^{2}+R_{xx}^{\text{Sigmoid}}\left(h-p-k\right)R_{xx}^{\text{Sigmoid}}\left(p-h-k\right)\right\}\right\}\\ & ={\left[\frac{M-\left|k\right|}{M}{\hat{R}}_{xx,\rho }^{TS}\left(k\right)\right]}^{2}\\ & +\underset{N\to \infty }{\lim}\frac{1}{2N+1}\sum_{n=-N}^{N}\exp\left(jnkT^{2}\cot\rho \right)\underset{N\to \infty }{\lim}\frac{1}{2N+1}\sum_{n=-N}^{N}\exp\left(jnkT^{2}\cot\rho \right)\\ & \cdot \left\{\frac{1}{M^{2}}\sum_{h=0}^{M-1-\left|k\right|}\sum_{p=0}^{M-1-\left|k\right|}\left\{{\left[R_{xx}^{\text{Sigmoid}}\left(h-p\right)\right]}^{2}+R_{xx}^{\text{Sigmoid}}\left(h-p-k\right)R_{xx}^{\text{Sigmoid}}\left(p-h-k\right)\right\}\right\} \end{array}$$

According to the Equations (A19) and (A20), we can obtain as follows:

(A21) $$\begin{array}{cc} \mathrm{Var}\left[{\hat{\hat{R}}}_{xx,\rho }^{TS}\left(k\right)\right] & =\underset{N\to \infty }{\lim}\frac{1}{2N+1}\sum_{n=-N}^{N}\exp\left(jnkT^{2}\cot\rho \right)\underset{N\to \infty }{\lim}\frac{1}{2N+1}\sum_{n=-N}^{N}\exp\left(jnkT^{2}\cot\rho \right)\\ & \cdot \left\{\frac{1}{M}\sum_{i=-\left(M-1-\left|k\right|\right)}^{M-1-\left|k\right|}\left[1-\frac{\left|k\right|+\left|i\right|}{M}\right]\left\{{\left[R_{xx}^{\mathrm{Sigmoid}}\left(i\right)\right]}^{2}+R_{xx}^{\mathrm{Sigmoid}}\left(i-k\right)R_{xx}^{\mathrm{Sigmoid}}\left(i+k\right)\right\}\right\} \end{array}$$

When M→∞, we can obtain $\mathrm{Var}\left[{\hat{\hat{R}}}_{xx,\rho }^{TS}\left(k\right)\right]=0$.

In summary, according to the $\mathrm{bia}\left[{\hat{\hat{R}}}_{xx,\rho }^{TS}\left(k\right)\right]=0$ and $\mathrm{Var}\left[{\hat{\hat{R}}}_{xx,\rho }^{TS}\left(k\right)\right]=0$, we may draw the conclusion that the estimator of the TS-FC is the the asymptotic consistent estimation.

### Appendix B.2. Unbiasedness and Consistency of the TS-FPSD

Let the estimator of the TS-FPSD be:

(A22) $${\hat{P}}_{xx,\rho }^{TS}\left(w\right)=A_{-\rho }{\tilde{F}}^{\rho }\left[{\hat{\hat{R}}}_{xx,\rho }^{TS}\left(k\right)\right]\left(w\right)\exp\left(-j\left(w^{2}/2T^{2}\right)\cot\rho \right)\;$$

Then:

(A23) $$\begin{array}{cc} E\left[{\hat{P}}_{xx,\rho }^{TS}\left(w\right)\right] & =E\left[A_{-\rho }{\tilde{F}}^{\rho }\left[{\hat{\hat{R}}}_{xx,\rho }^{TS}\left(k\right)\right]\left(w\right)\exp\left(-j\left(w^{2}/2T^{2}\right)\cot\rho \right)\right]\\ & =A_{-\rho }{\tilde{F}}^{\rho }\left[E\left[{\hat{\hat{R}}}_{xx,\rho }^{TS}\left(k\right)\right]\right]\left(w\right)\exp\left(-j\left(w^{2}/2T^{2}\right)\cot\rho \right) \end{array}$$

According to the Equation (A17), Equation (A23) can be rewritten as:

(A24) $$\begin{array}{cc} E\left[{\hat{P}}_{xx,\rho }^{TS}\left(w\right)\right] & =A_{-\rho }{\tilde{F}}^{\rho }\left[\frac{M-\left|k\right|}{M}{\hat{R}}_{xx,\rho }^{TS}\left(k\right)\right]\left(w\right)\exp\left(-j\left(w^{2}/2T^{2}\right)\cot\rho \right)\\ & =A_{-\rho }{\tilde{F}}^{\rho }\left[{\hat{R}}_{xx,\rho }^{TS}\left(k\right)\right]\left(w\right)\exp\left(-j\left(w^{2}/2T^{2}\right)\cot\rho \right)\\ & +A_{-\rho }{\tilde{F}}^{\rho }\left[-\frac{\left|k\right|}{M}{\hat{R}}_{xx,\rho }^{TS}\left(k\right)\right]\left(w\right)\exp\left(-j\left(w^{2}/2T^{2}\right)\cot\rho \right)\\ & =P_{xx,\rho }^{TS}\left(w\right)+W_{xx,\rho }^{TS}\left(w\right) \end{array}$$

where $W_{xx,\rho }^{TS}\left(w\right)$ denotes the TS-FPSD of the $-\frac{\left|k\right|}{M}{\hat{R}}_{xx,\rho }^{TS}\left(k\right)$.

For a given |k| value and as M→∞, $W_{xx,\rho }^{TS}\left(w\right)=0$, i.e.:

(A25) $$E\left[{\hat{P}}_{xx,\rho }^{TS}\left(w\right)\right]=P_{xx,\rho }^{TS}\left(w\right)\;$$

Thus, $\mathrm{bia}\left[{\hat{P}}_{xx,\rho }^{TS}\left(w\right)\right]=0$, and the estimator of the TS-FPSD is the asymptotic unbiased estimation.

According to the definition of the consistency, we can obtain:

(A26) $$\begin{array}{cc} \mathrm{Var}\left[{\hat{P}}_{xx,\rho }^{TS}\left(w\right)\right] & =E\left\{{\left[{\hat{P}}_{xx,\rho }^{TS}\left(w\right)-E\left[{\hat{P}}_{xx,\rho }^{TS}\left(w\right)\right]\right]}^{2}\right\}\\ & =E\left\{{\left[{\hat{P}}_{xx,\rho }^{TS}\left(w\right)\right]}^{2}\right\}-{\left\{E\left[{\hat{P}}_{xx,\rho }^{TS}\left(w\right)\right]\right\}}^{2} \end{array}$$

Equation (A26) may involve the fourth moment problem, which is difficult to solve directly. Therefore, we may compute the covariance of the ${\hat{P}}_{xx,\rho }^{TS}\left(w\right)$ with $w_{1}$ and $w_{2}$ as follows:

(A27) $$\begin{array}{cc} \mathrm{Cov}\left[{\hat{P}}_{xx,\rho }^{TS}\left(w_{1}\right),{\hat{P}}_{xx,\rho }^{TS}\left(w_{2}\right)\right] & =E\left\{\left[{\hat{P}}_{xx,\rho }^{TS}\left(w_{1}\right)-E\left\{{\hat{P}}_{xx,\rho }^{TS}\left(w_{1}\right)\right\}\right]\left[{\hat{P}}_{xx,\rho }^{TS}\left(w_{2}\right)-E\left\{{\hat{P}}_{xx,\rho }^{TS}\left(w_{2}\right)\right\}\right]\right\}\\ & =E\left\{{\hat{P}}_{xx,\rho }^{TS}\left(w_{1}\right){\hat{P}}_{xx,\rho }^{TS}\left(w_{2}\right)\right\}-E\left\{{\hat{P}}_{xx,\rho }^{TS}\left(w_{1}\right)\right\}E\left\{{\hat{P}}_{xx,\rho }^{TS}\left(w_{2}\right)\right\} \end{array}$$

Where:

(A28) $$\begin{array}{c} E\left\{{\hat{P}}_{xx,\rho }^{TS}\left(w_{1}\right){\hat{P}}_{xx,\rho }^{TS}\left(w_{2}\right)\right\}\\ =E\left\{\left[A_{-\rho }{\tilde{F}}^{\rho }\left[{\hat{\hat{R}}}_{xx,\rho }^{TS}\left(k\right)\right]\left(w_{1}\right)\exp\left(-j\left(w_{1}^{2}/2T^{2}\right)\cot\rho \right)\right]\left[A_{-\rho }{\tilde{F}}^{\rho }\left[{\hat{\hat{R}}}_{xx,\rho }^{TS}\left(k\right)\right]\left(w_{2}\right)\exp\left(-j\left(w_{2}^{2}/2T^{2}\right)\cot\rho \right)\right]\right\}\\ =\left[A_{-\rho }{\tilde{F}}^{\rho }\left\{E\left[{\hat{\hat{R}}}_{xx,\rho }^{TS}\left(k\right)\right]\right\}\left(w_{1}\right)\exp\left(-j\left(w_{1}^{2}/2T^{2}\right)\cot\rho \right)\right]\left[A_{-\rho }{\tilde{F}}^{\rho }\left\{E\left[{\hat{\hat{R}}}_{xx,\rho }^{TS}\left(k\right)\right]\right\}\left(w_{2}\right)\exp\left(-j\left(w_{2}^{2}/2T^{2}\right)\cot\rho \right)\right] \end{array}$$

Let $w_{1}=w_{2}$; we can obtain:

(A29) $$\mathrm{Cov}\left[{\hat{P}}_{xx,\rho }^{TS}\left(w_{1}\right),{\hat{P}}_{xx,\rho }^{TS}\left(w_{1}\right)\right]=\mathrm{Var}\left[{\hat{P}}_{xx,\rho }^{TS}\left(w\right)\right]\;$$

According to Equation (A17), Equation (A28) can be rewritten as:

(A30) $$\begin{array}{l} E\left\{{\hat{P}}_{xx,\rho }^{TS}\left(w_{1}\right){\hat{P}}_{xx,\rho }^{TS}\left(w_{2}\right)\right\}\\ =\left[A_{-\rho }{\tilde{F}}^{\rho }\left\{\frac{M-\left|k\right|}{M}{\hat{R}}_{xx,\rho }^{TS}\left(k\right)\right\}\left(w_{1}\right)\exp\left(-j\left(w_{1}^{2}/2T^{2}\right)\cot\rho \right)\right]\\ \cdot \left[A_{-\rho }{\tilde{F}}^{\rho }\left\{\frac{M-\left|k\right|}{M}{\hat{R}}_{xx,\rho }^{TS}\left(k\right)\right\}\left(w_{2}\right)\exp\left(-j\left(w_{2}^{2}/2T^{2}\right)\cot\rho \right)\right] \end{array}$$

For a given |k| value and as M→∞, Equation (A30) can be rewritten as:

(A31) $$E\left\{{\hat{P}}_{xx,\rho }^{TS}\left(w_{1}\right){\hat{P}}_{xx,\rho }^{TS}\left(w_{2}\right)\right\}=P_{xx,\rho }^{TS}\left(w_{1}\right)P_{xx,\rho }^{TS}\left(w_{2}\right)\;$$

Substituting Equation (A31) into Equation (A27), we can obtain the covariance of the ${\hat{P}}_{xx,\rho }^{TS}\left(w\right)$ as:

(A32) $$\mathrm{Cov}\left[{\hat{P}}_{xx,\rho }^{TS}\left(w_{1}\right),{\hat{P}}_{xx,\rho }^{TS}\left(w_{2}\right)\right]=P_{xx,\rho }^{TS}\left(w_{1}\right)P_{xx,\rho }^{TS}\left(w_{2}\right)-E\left\{{\hat{P}}_{xx,\rho }^{TS}\left(w_{1}\right)\right\}E\left\{{\hat{P}}_{xx,\rho }^{TS}\left(w_{2}\right)\right\}\;$$

According to Equations (A26)–(A29) and Equations (A31)–(A32), we can get:

(A33) $$\mathrm{Var}\left[{\hat{P}}_{xx,\rho }^{TS}\left(w\right)\right]={\left[P_{xx,\rho }^{TS}\left(w\right)\right]}^{2}-{\left[E\left\{{\hat{P}}_{xx,\rho }^{TS}\left(w\right)\right\}\right]}^{2}=0\;$$

In summary, for $\mathrm{bia}\left[{\hat{P}}_{xx,\rho }^{TS}\left(w\right)\right]=0$ and $\mathrm{Var}\left[{\hat{P}}_{xx,\rho }^{TS}\left(w\right)\right]=0$, we may draw the conclusion that the estimator of the TS-FPSD is the asymptotic consistent estimation.
